# Mutations in Global Regulators Lead to Metabolic Selection during Adaptation to Complex Environments

**DOI:** 10.1371/journal.pgen.1004872

**Published:** 2014-12-11

**Authors:** Gerda Saxer, Michael D. Krepps, Eric D. Merkley, Charles Ansong, Brooke L. Deatherage Kaiser, Marie-Thérèse Valovska, Nikola Ristic, Ping T. Yeh, Vittal P. Prakash, Owen P. Leiser, Luay Nakhleh, Henry S. Gibbons, Helen W. Kreuzer, Yousif Shamoo

**Affiliations:** 1 Department of BioSciences, Rice University, Houston, Texas, United States of America; 2 United States Army Edgewood Chemical Biological Center, Aberdeen Proving Ground, Maryland, United States of America; 3 EXCET, Inc, Springfield, Virginia, United States of America; 4 Pacific Northwest National Laboratory, Richland, Washington, United States of America; 5 Department of Computer Science, Rice University, Houston, Texas, United States of America; 6 Center for Microbial Genetics and Genomics, Northern Arizona University, Flagstaff, Arizona, United States of America; Université Paris Descartes, INSERM U1001, France

## Abstract

Adaptation to ecologically complex environments can provide insights into the evolutionary dynamics and functional constraints encountered by organisms during natural selection. Adaptation to a new environment with abundant and varied resources can be difficult to achieve by small incremental changes if many mutations are required to achieve even modest gains in fitness. Since changing complex environments are quite common in nature, we investigated how such an epistatic bottleneck can be avoided to allow rapid adaptation. We show that adaptive mutations arise repeatedly in independently evolved populations in the context of greatly increased genetic and phenotypic diversity. We go on to show that weak selection requiring substantial metabolic reprogramming can be readily achieved by mutations in the global response regulator *arcA* and the stress response regulator *rpoS.* We identified 46 unique single-nucleotide variants of *arcA* and 18 mutations in *rpoS*, nine of which resulted in stop codons or large deletions, suggesting that subtle modulations of ArcA function and knockouts of *rpoS* are largely responsible for the metabolic shifts leading to adaptation. These mutations allow a higher order metabolic selection that eliminates epistatic bottlenecks, which could occur when many changes would be required. Proteomic and carbohydrate analysis of adapting *E. coli* populations revealed an up-regulation of enzymes associated with the TCA cycle and amino acid metabolism, and an increase in the secretion of putrescine. The overall effect of adaptation across populations is to redirect and efficiently utilize uptake and catabolism of abundant amino acids. Concomitantly, there is a pronounced spread of more ecologically limited strains that results from specialization through metabolic erosion. Remarkably, the global regulators *arcA* and *rpoS* can provide a “one-step” mechanism of adaptation to a novel environment, which highlights the importance of global resource management as a powerful strategy to adaptation.

## Author Summary

Changing environmental conditions are the norm in biology. However, understanding adaptation to complex environments presents many challenges. For example, adaptation to resource-rich environments can potentially have many successful evolutionary trajectories to increased fitness. Even in conditions of plenty, the utilization of numerous but novel resources can require multiple mutations before a benefit is accrued. We evolved two bacterial species isolated from the gut of healthy humans in two different, resource-rich media commonly used in the laboratory. We anticipated that under weak selection the population would evolve tremendous genetic diversity. Despite such a complex genetic background we were able to identify a strong degree of parallel evolution and using a combination of population proteomic and population genomic approaches we show that two global regulators, *arcA* and *rpoS,* are the principle targets of selection. Up-regulation of the different metabolic pathways that are controlled by these global regulators in combination with up-regulation of transporters that transport nutrients into the cell revealed increased use of the novel resources. Thus global regulators can provide a one-step model to shift metabolism efficiently and provide rapid a one-step reprogramming of the cell metabolic profile.

## Introduction

Adaptation to novel environments can proceed either through many mutations with small effects or through few mutations with large effects [1]. Adaptation to complex environments is the norm in biology, but a clear understanding of the adaptive processes employed by organisms in ecologically diverse environments is challenging. Ecological complexity can arise from increased species diversity, spatial or temporal heterogeneity or different resources. The availability of countless resources in complex environments can lead to a rapid and substantial increase in genetic diversity that can obscure broader biochemical principles of adaptation. For example, if resources are varied and plentiful, the population can accumulate mutations in genes that are not essential for survival in the selective environment [2], [3]. Thus, in nutrient-rich environments, specialists with narrower niches can persist by using alternative resources without necessarily improving their fitness relative to the ancestor. Adaptation then occurs through specialization via fitness improvements or via metabolic erosion possibly without fitness improvements relative to the ancestor [3]–[5]. We hypothesized that, despite the tremendous opportunities for increased genetic diversity under conditions of plenty, consistent adaptive responses could be observed as parallel evolution across and within independently evolved populations. We further reasoned that such general and consistent adaptive responses could be driven by global metabolic regulators to provide an efficient reprogramming of metabolic networks with a minimal number of steps.

Experimental evolution of model organisms under novel conditions is a versatile approach for understanding the evolutionary dynamics of adaptation and the functional constraints that shape the physiological evolvability of an organism. Typically, microbial model organisms are selected for adaptation to a single or a few distinct resources [6]–[11], to antibiotics [12], [13], or to temperature [14], [15]. Experiments in such relatively simple selective environments have shown that during adaptation to a single resource, the evolving population typically climbs a single-peak fitness landscape in an incremental manner with diminishing returns epistasis [16]–[18]. Even in such simple environments, resource partitioning or spatial or temporal heterogeneity can lead to the evolution of different specialists and complex ecological interactions [10], [11], [19]–[26].

To better understand the evolutionary and adaptive dynamics in ecologically complex environments, we focused on resource availability and conducted selection experiments in very nutrient-rich conditions. Unlike adaptation to a single limiting resource that is often conceptualized as a single fitness peak, a wealth of resources will potentially present abundant peaks in the fitness landscape. Because resources may differ only slightly, the selection differences can be very small and are reflected as very modest fitness peaks. As a consequence, selection will be weak and lead to an increase in genetic variation through the accumulation of mutations, though the fixation of any specific mutation would be unlikely. Identifying adaptive mutations in such genetically diverse populations can be difficult. However, we reasoned that adaptive mutations should evolve repeatedly in independent populations, while neutral or deleterious mutations should not show any discernible degree of parallelism. Parallel evolution has been readily observed in nature regardless of the ecological complexity [11], [27], [28]. Such adaptive, phenotypic convergence can be based on different underlying genetic changes, such that adaptive, parallel changes can occur in the same gene, or in different genes of the same pathway or functional group [12], [15].

In an environment where selection is weak and selective differences are small, it is hard to imagine a scenario where an individual can quickly accumulate mutations along a multi-protein pathway that will lead to increased fitness. Instead, mutations in regulatory genes such as transcriptional or translational regulators that can simultaneously affect many operons or entire pathways could produce much larger benefits and circumvent potential complications from epistatic interactions among different mutations. One example of a gene with such large pleiotropic effects is the global stress response regulator *rpoS*, which is activated during late exponential and stationary phase [29]. Mutations in *rpoS* are often among the first mutations to evolve during experimental evolution of *E. coli* [30] and have been routinely observed in different selective conditions [31]–[33]. These mutations lead to changes in the stress response and nutrient acquisition [34], change the stress induced mutation rates [35], [36] and increase long-term viability [37]. Knocking out *rpoS* leads to a down-regulation of the starvation stress response and efflux pumps, and to increased nutrient efficiency via the up-regulation of proteins such as porins. The trade-off of stress resistance and nutritional competence was termed the SPANC balance (self preservation and nutritional competence) by Ferenci [34]. While the prevalence of *rpoS* knockout mutants is low in wild isolates [38], considerable variation in *rpoS* expression has been observed among wild strains [39]. We therefore hypothesized that mutations in global regulators could be especially beneficial in ecologically complex, nutrient-rich environments that induce weak selection.

**Figure 1:**
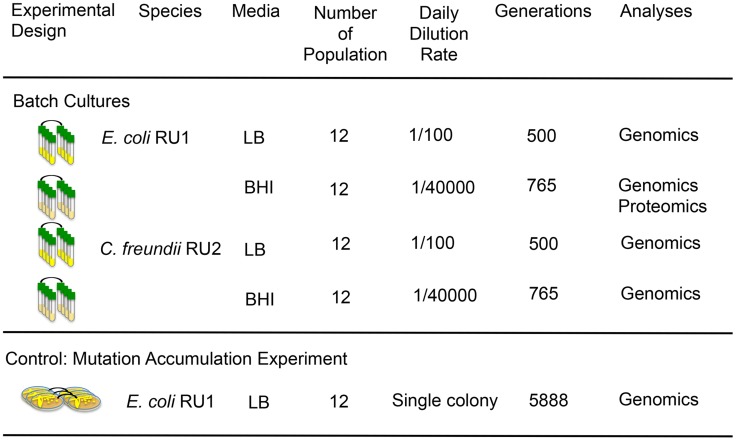
Population genomics and population proteomics were used to identify the biochemical basis for phenotypic convergence and parallel evolution during adaptation to novel resource rich environments. Clones of two species, *E. coli* RU1 and *C. freundii* RU2, were isolated *de novo* from human stool. Single clones were used to inoculate twelve replicated populations and evolved in LB or BHI for 500 or 765 generations respectively by daily transfers into fresh media. Adaptive mutations were identified as mutations that evolved repeatedly across species and media. We performed a mutation accumulation experiment by transferring a single colony of twelve replicated lines for 200 days as control experiment. The relaxed selection allowed the random accumulation of mutations, and makes the parallel evolution of adaptive mutations unlikely.

In contrast to previous experiments where laboratory adapted strains were evolved in rich media commonly used in the laboratory [40], we isolated naïve strains from their natural habitat, the gut of healthy humans, and used rich media as novel, selective environments. In the gut, *E. coli* and *C. freundii* interact with hundreds of other strains as well as their human host. More than 90% of these commensal gut bacteria are anaerobes, which convert non-digestible complex carbohydrates into short-chain fatty acids and produce the simple mono- and di-saccharides favored by *E. coli* [41], [42]. In return, facultative anaerobes like *E. coli* and *C. freundii* play an important role in maintaining a low-oxygen environment. We chose two complex media (BBL BHI and LB Miller) that differed primarily in the composition and amounts of amino acids, vitamins and carbohydrates (S1 Text). In addition to the populations selected in complex media (two genotypes in two environments resulting in four treatments, Fig. 1), we also performed a control experiment, where we reduced selection as much as possible by daily bottlenecking the population to a single cell, the approach commonly used for mutation accumulation (MA) experiments [43]–[45]. In mutation accumulation experiments, independently evolved lines are expected to accumulate a random set of mutations with little to no parallel evolution.

We used a powerful combination of population proteomics and population genomics to reveal phenotypic convergence to identify potential biochemical mechanisms of adaptation. Despite the complexities imposed by the tremendous amount of underlying genetic diversity accumulated during adaptation to complex nutrient rich environments, we identified clear genomic signatures of adaptation across and within independently evolved populations. Strikingly, changes in the global regulators *arcA* and *rpoS* evolved consistently, while changes in other global regulators were largely absent. Subsequent proteomic and carbohydrate analysis of populations adapting to BHI showed increased abundance of enzymes associated with the TCA cycle and amino acid metabolism to make use of abundant amino acids, resulting in the secretion of the polyamine putrescine as a nitrogen sink. Thus in complex media, where the adaptive landscape is relatively flat and has many potential modest peaks requiring many changes to produce a substantive increase in fitness, the “go to” strategy may be to use global regulators such as *arcA* and *rpoS* to overcome epistasis by changing the regulation of whole metabolic pathways in a coordinated manner. This allows populations to rapidly reprogram resource utilization and to adapt to complex fitness landscapes in a much smaller number of moves.

## Results

### Parallel adaptation to complex environments is readily observed across populations despite a large amount of genetic variation

We isolated *E. coli* RU1 and *C. freundii* RU2 (S1 Figure) *de novo* from the gut flora of healthy humans using only two overnight growths on agar to reduce any selection prior to our adaptation experiment. For each species, 12 populations were established and allowed to adapt over a minimum of 500 generations to media and conditions that were very rich in resources and substantially different than the environment of the human gut (Fig. 1). We chose LB and BHI as novel environments since they are likely to be very different from the gut resource base, but still support robust growth of both ancestral strains (S1 Table).

Over the course of the selection experiments, we observed modest but significant changes in various fitness components, consistent with adaptation under weak selection conditions. While lag time decreased in most treatments, maximum growth remained constant in LB and decreased in BHI (S1 Table). In all but one treatment, LB-evolved *C. freundii*, the populations significantly increased their stationary phase density (OD_600_) indicating enhanced abilities to utilize the resources efficiently. While we observed significant changes, the differences between ancestor and evolved populations were modest and consistent with permissive environments inducing weak selection pressures (S1 Table, S1 Text).

**Figure 2:**
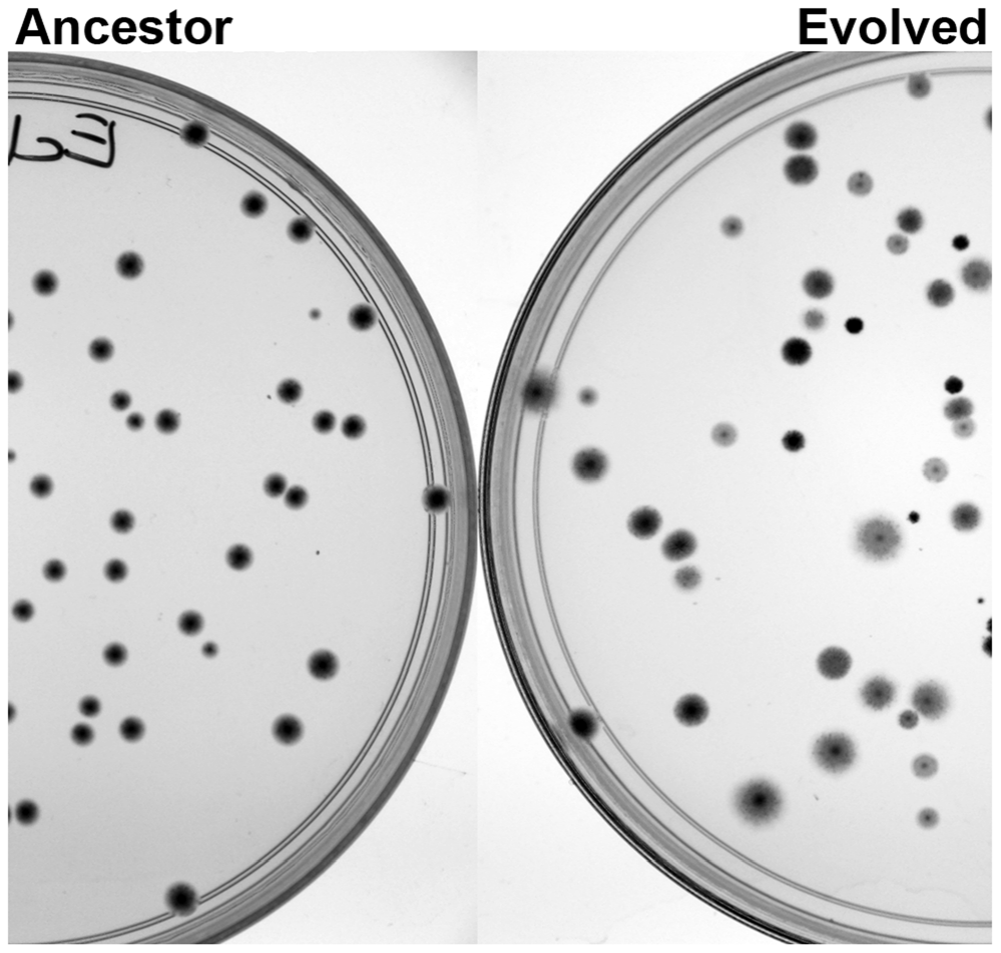
The underlying genetic diversity of the adapted populations is readily observed as phenotypic diversity, when plated on tetrazolium arabinose plates. When arabinose is provided as the carbon source, the diversity of genotypes in the adapted populations is seen as a marked increase in phenotypic diversity. Colony size, morphology and the ability to use arabinose (as indicated by the darkness of the colony) varied widely in the adapted populations compared to the ancestor.

As a consequence of weak selection, considerable genetic variation evolved over the course of our experiment. This was evident both at the phenotypic as well as the genotypic level. We observed considerable phenotypic variation in colony size and in the ability to utilize arabinose (Fig. 2), in redox activity, in exopolysaccharide content and loss of motility (S2A-D Figure, respectively). Interestingly, evolved *E. coli* populations had at least one colony among the 8 colonies assessed per population that lost motility, but only one single *C. freundii* colony out of all the colonies assessed (two sets of 96 colonies in total) lost motility (S1 Text).

**Figure 3:**
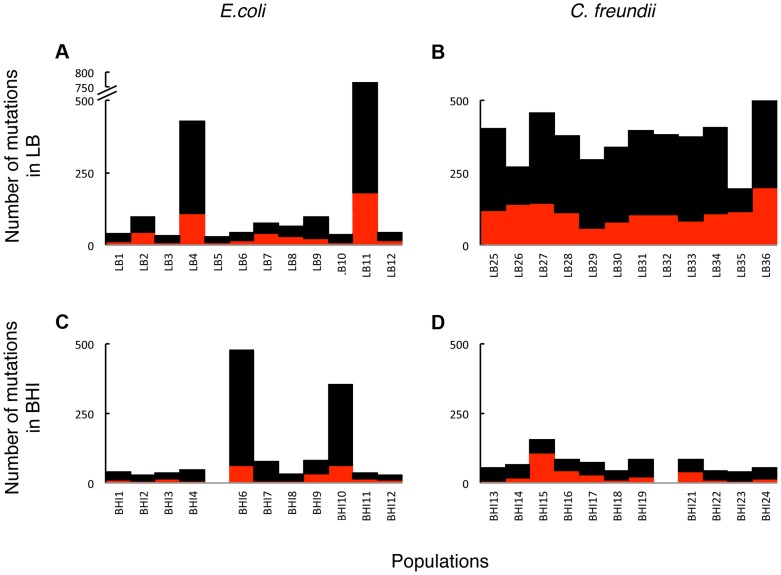
The total number of mutations in coding regions was substantial in the evolved populations. The accumulation of hundreds of mutations is consistent with weak selection, where many mutations can have small consequences for the fitness of organism. Overall, more mutations evolved in LB (A, B) than in BHI (C, D), both in the *E. coli* (A, C) and the *C. freundii* (B, D) populations. Two LB- and two BHI-evolved *E. coli* populations acquired considerably more mutations than the remaining populations evolved in the same media, suggesting that these populations evolved to become mutators. Total number of mutations in coding regions (black) and non-synonymous mutations (red) are shown for each population. Populations BHI5 and BHI20 were not included in the genomic analyses.

To assess the evolved genetic variation and identify adaptive mutations, we sequenced the evolved populations and identified mutations in coding regions that occurred at a minimum frequency of 0.05 in a population. Two BHI-evolved populations (*E.coli* BHI5 and *C. freundii* BHI20) could not be aligned properly and were omitted from further genomic analyses. The number of mutations ranged from 29 to 725 per population. The number of mutations per population did not differ significantly among the *E. coli* populations evolved in LB or BHI (Fig. 3; Table 1). Two populations evolved to become mutators in each environment (LB4, LB11, BHI6, and BHI10, S1 Text). If the mutator populations are excluded, the average number of mutations between the LB and BHI-evolved populations was reduced, although there was still no significant difference in the number of mutations across environments. In contrast, the LB-evolved *C. freundii* populations accumulated significantly more mutations than the BHI-evolved populations. Overall, the number of mutations differed significantly both between media and species (Full factor ANOVA with Media and Strain as fixed factors: Media F_1,42_ = 15.1, p = 0.0004, Species F_1,42_ = 4.5, p = 0.039, Media×Species F_1,42_ = 9.5, p = 0.0036). While synonymous mutations can have fitness effects [46], [47], we focused our analyses on non-synonymous mutations, which include SNPs, insertions, and deletions. The number of non-synonymous mutations ranged from 5 to 198 in a population, with more mutations arising in the LB than in BHI in the *E. coli* population (Fig. 3, Table 1). Excluding the mutator populations reduced the average non-synonymous mutations per population further (LB: 21±15, BHI: 12±7). Among the *C. freundii* populations, the average number of non-synonymous mutations was significantly higher in the LB-evolved populations than in the BHI-evolved populations. Again, we observed significant differences among media and species (Media  = F_1,42_ = 23.8, p<0.0001, Species: F_1,42_ = 13.2, p = 0.0007, Media×Species: F_1,42_ = 9.4, p = 0.0037).

**Table 1:** Average number of mutations in the four different treatments.

Species	Media	Total mutations^1^	Total mutations excluding mutator populations	Non-synonymous mutations^2^	Non-synonymous mutations excluding mutator populations^2^
*E. coli*	LB	147±141	57±18	41±33	21±15
	BHI	112±102	45±14	21±14	12±7
*C. freundii*	LB	370±57		144±23	
	BHI	72±22		27±20	

1 mean and 95% CI.

2 includes SNPs, insertions and deletions.

**Figure 4:**
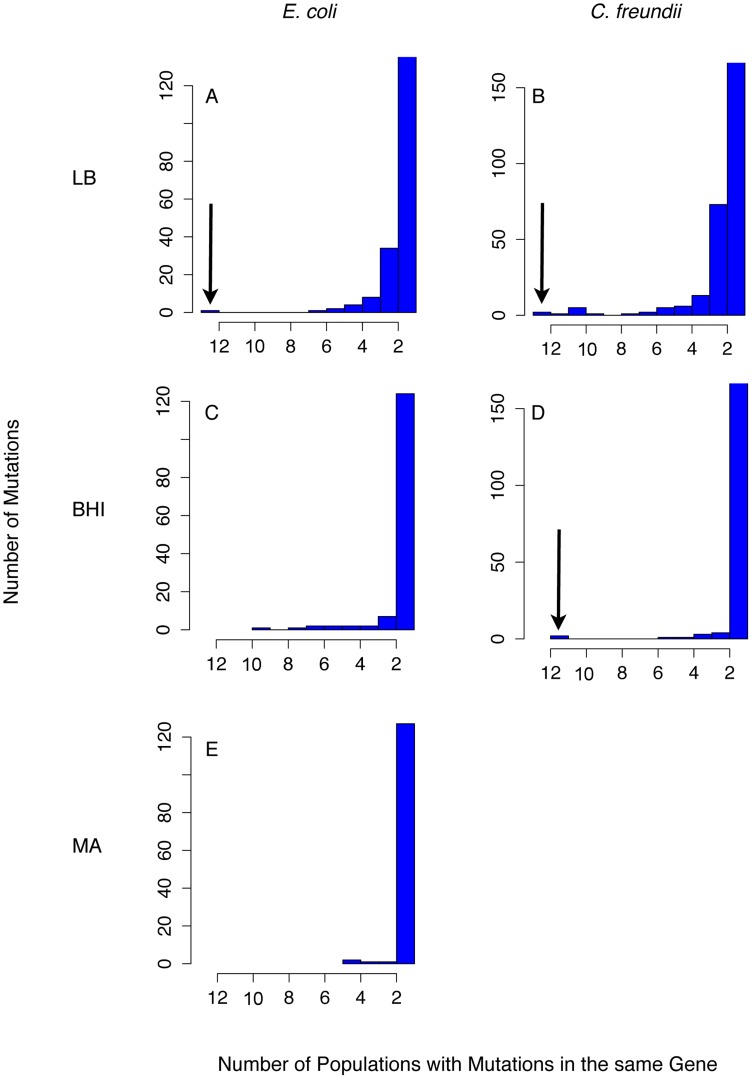
Parallel evolution was observed as mutations that evolved in many populations. Only a few genes acquired mutations consistently across species and media (indicated by the arrow). Importantly, none of these genes acquired mutations in the control experiment, the mutation accumulation lines (MA, (E)). As expected from our conditions of weak selection, most mutations occurred only in a few populations. We analyzed 24 LB-evolved (A and B), 22 BHI-evolved populations (C and D) and 12 mutation accumulation lines as a control experiment (MA) (E). The number of mutations found in only one population was capped at 120 (LB) or 150 (BHI) to better discern the number of genes that evolved in parallel.

**Figure 5:**
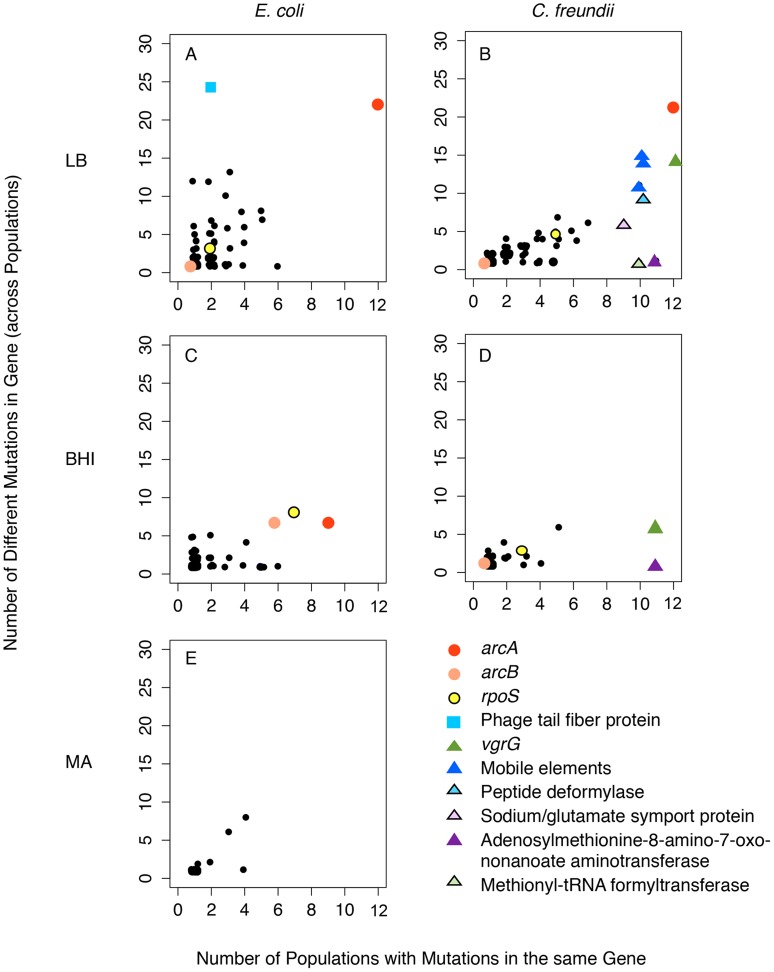
Mutations in *arcA* and *rpoS* evolved repeatedly both within and among *E. coli* populations evolved in LB (A) and BHI (C), and *C. freundii* populations evolved in LB (B) and BHI (D). The number of mutations observed in the evolved populations is plotted over the number of populations with mutations in that gene. The scatterplots show all the genes with mutations for the twelve populations evolved in one environment (black circles). Selected genes that evolved in parallel are identified and marked individually, following the legend. None of these evolved genes had mutations in the mutation accumulation lines of *E. coli* evolved on LB agar (E).

The accumulation of largely non-adaptive mutations complicates the identification of adaptive changes within a single, polymorphic population. However, we expected that important adaptive trajectories would exist across independently evolved populations. Therefore, we focused our analyses on parallel, non-synonymous mutations that evolved consistently across populations, both within and across species and media. Most mutations occurred in only one or a few populations, consistent with the presence of large non-adaptive genetic variation (Fig. 4). Strong parallel evolution across environments and species occurred in the global regulator *arcA*, which acquired mutations in all 24 LB-evolved populations and in nine of eleven BHI-evolved *E. coli* populations (Fig. 5). The probability that mutations evolved in the same gene in 24 independently evolved populations at random is very small considering that *E. coli* RU1 and *C. freundii* RU2 had 4565 and 5068 annotated genes, respectively (p = (1/4565)^12^ *(1/5068)^12^) and suggests that these mutations are adaptive. Surprisingly, *C. freundii* adaptation to BHI did not implicate *arcA*. The second most commonly mutated gene was the global stress response regulator *rpoS* [29], [48], which had mutations in nine of 23 *E. coli* and eight of 23 *C. freundii* populations. None of the mutation accumulation lines had mutations in *arcA* or *rpoS*.

Besides *arcA* and *rpoS*, only a few other genes acquired mutations in replicated populations, and unlike *arcA* and *rpoS*, these other mutations occurred only within a treatment and not across species and selective environment. No other mutation evolved with any degree of parallelism in the *E. coli* populations. Among the *C. freundii* populations, mutations in a gene encoding the Valine-Glycine Repeat Protein G, *vgrG*, a homolog to the tailspike of bacteriophage T4, and in a gene encoding adenosylmethionine-8-amino-7-oxononanoate aminotransferase, an enzyme involved in biotin biosynthesis, occurred in almost all evolved populations in both selective environments, while mutations in different mobile elements, in the peptide deformylase, the methionyl-tRNA formyltransferase and the sodium/glutamate symport protein were only common among the LB-evolved *C. freundii* populations (for further details see S1 Text).

### Mutations in *arcA* and *rpoS* drive adaptation to the complex selective environments

**Figure 6:**
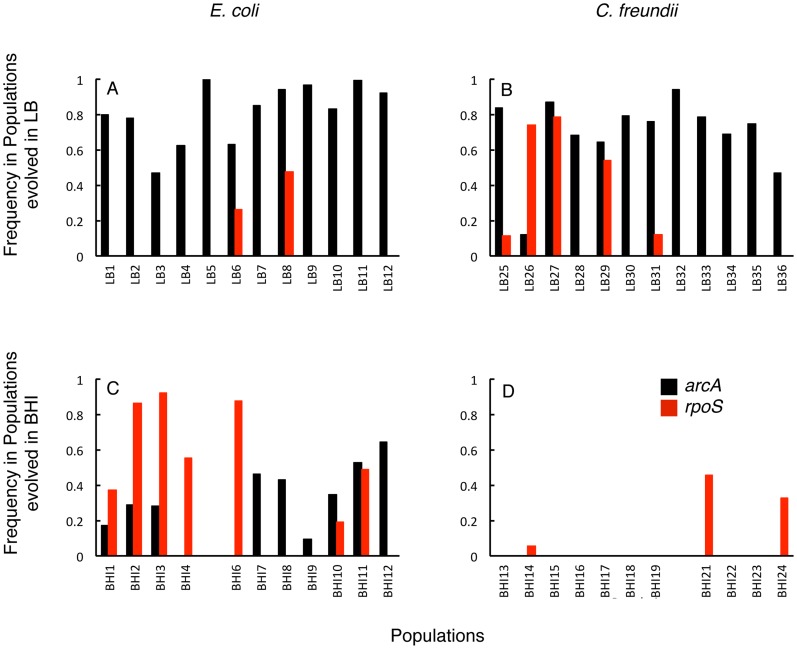
Frequencies of *arcA* and *rpoS* mutants in the evolved populations *arcA* mutations (black bars) reached high frequencies in all LB-evolved populations (A, B) and reach fixation in LB5, while *rpoS* mutations (red) were more common in BHI-evolved populations (C, D). The frequencies represent the total frequencies of all *arcA* or respectively, identified in a particular population.

The highly parallel evolution of mutations in *arcA* and *rpoS* combined with their global effects suggests that these mutations are driving adaptation in these complex selective environments. Mutations in these genes were very common with multiple different alleles co-occurring within the same population. The cumulative frequencies of *arcA* mutations in particular reached high frequencies in LB (average 0.75±0.08 (mean and 95%CI) across 24 populations (Fig. 6). In only one population did we observe the fixation of a single *arcA* mutation (LB5).

**Figure 7:**
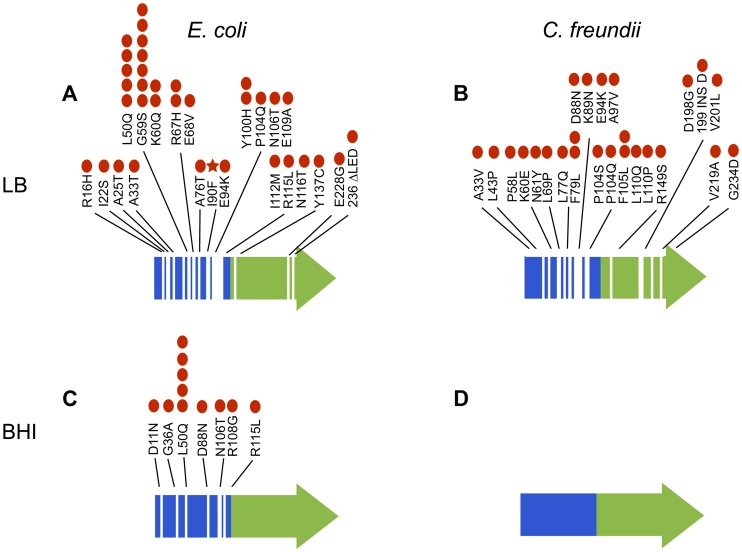
Mutations in *arcA* evolved repeatedly and with remarkable diversity both within and among populations of *E. coli* evolved in LB (A) and BHI (C) and *C. freundii* populations evolved in LB (B) and BHI (D). Specific mutations to *arcA* identified in the evolved populations are indicated. The red dots represent the number of populations with that specific mutation (out of twelve LB and eleven BHI populations for each strain). The red star indicates the mutation that was fixed in LB5. No mutations in *arcA* were identified in the BHI-evolved *C. freundii* populations. The receiver domain that includes the site of phosphorylation (Asp-54) is indicated in blue and the DNA binding domain in green.

We observed 46 unique mutations in *arcA*, both within and among populations (Fig. 7). Strikingly, none of these mutations introduced a stop codon or a frame shift; 44 of these 46 unique mutations were non-synonymous substitutions, one mutation resulted in a C-terminal deletion of three amino acids, and one mutation was an insertion of one amino acid. To independently confirm some of the mutations identified from population genomics, we directly sequenced *arcA* from eight single colonies isolated from six of the LB-evolved *E. coli* populations. We were able to confirm eleven of the 46 mutations identified in the whole population samples (L2: I122M, Y137C, I22S, L4: N116T, L6: R16H, A76T; L8: E94K; L10: A25T, G59S, L50Q; and L12: L50Q). In addition, we identified two new mutations (L8: G62D and 218ΔTPE; and L12: G62D) suggesting that our cutoff of 5% in the deep sequencing population analysis still missed many *arcA* variants. Each clone had only one mutation in *arcA*, suggesting that the one mutation was sufficient to achieve a beneficial effect.

The response regulator *arcA* is part of the two-component *arcAB* signal transduction system. The membrane bound sensor kinase ArcB phosphorylates ArcA (ArcA-P) in response to a variety of environmental challenges to maintain redox and metabolic homeostasis [49]–[51]. Mutations in the sensor kinase *arcB* could also affect the regulation of *arcA*. We therefore examined the whole genome sequencing data for mutations in the sensor kinase *arcB* and found mutations in six of the BHI-evolved (BHI1, BHI2, BHI3, BHI4, BHI9 and BHI10) and in one of the LB-evolved *E. coli* populations (LB8). Among the *C. freundii* populations, mutations in *arcB* were less frequent, with only one population evolving mutations in *arcB* in each environment (LB26 and BHI24). As with *arcA*, all mutations were non-synonymous, though one mutation did result in a frame shift. Importantly, neither *arcA* nor *arcB* evolved mutations in the mutation accumulation lines suggesting that changes in *arcAB* are under selection and not random.

The amount of variation in *rpoS* mutations was not quite as dramatic as in *arcA*, but substantial nonetheless. In the LB-evolved *E. coli* populations we predominately observed SNPs, while in the BHI-evolved *E. coli* populations mutations in *rpoS* resulted in stop codons, large deletions or frame shift mutations, suggesting a loss of function (S3 Figure). Among the five LB-evolved and three BHI-evolved *C. freundii* populations with mutations in *rpoS*, only one population (LB31) acquired a mutation resulting in a stop codon while the rest acquired substitutions. To confirm some of these mutations independently, we sequenced *rpoS* from eight single colony isolates for three of the LB-evolved populations (LB6, LB8 and LB12) and confirmed the A199T mutation in population LB8 as well as a new *rpoS* mutation (Y283C) in LB6.

Unlike *arcA*, we observed many loss of function mutations in *rpoS*, which is consistent with previous selection experiments where knock-out mutations evolved relatively rapidly under different selective conditions [31]–[33]. RpoS levels could also be attenuated through changes in the regulation of its expression. The expression of *rpoS* is repressed during exponential growth and activated upon starvation during late exponential and stationary phase using different transcriptional and translational mechanisms. Transcription of *rpoS* is up-regulated through *spoT*/(p)ppGpp and BarA/UvrY and repressed by *arcAB*, while translation is up-regulated by two small RNAs, DsrA and RprA and repressed by a third small RNA ArcZ [29]. Presumably, mutations in any of these genes could also affect the up-regulation of *rpoS* and lead to reduced expression, resulting in similar phenotypes as the knockout mutants. We did not observe any mutations in *spoT,* though mutations in this gene evolve readily in minimal media [6], [52], [53]. Four of the LB-evolved *C. freundii* populations (LB27, LB32, LB35, LB36), however, had a frame shift mutation in *barA/uvrY*. Three of these populations did not have a mutation in *rpoS*, suggesting that the effect of loss of function mutations in *barA/uvrY* could lead to reduced transcription of *rpoS* and result in a similar phenotype to *rpoS* knockout mutations. Mutations in the small RNAs DsrA and RprA could also lead to reduced translation of *rpoS* and result in a similar phenotype as *rpoS* knockout mutants. We looked for mutations in DsrA and RprA in *E. coli* and did not detect any mutations in these small RNAs. While we also searched for the small RNAs in *C. freundii* using sequences retrieved from *Citrobacter*, we were unable to locate the two small RNAs in our reference genome.

Linking mutations to functional changes across regulatory sequences is more difficult as it requires excellent annotation and understanding of the transcriptional regulators. Nonetheless, it is certainly likely that changes in regulatory regions could provide adaptive changes. To test whether *arcA* or *rpoS* expression were altered by mutations within their regulatory regions, we examined the 500 nucleotides preceding the start codons of these two genes and found no mutation in any of the evolved populations or the MA lines.

### Evolved populations showed improved utilization of abundant amino acids through up-regulation of enzymes in the TCA cycle

**Figure 8:**
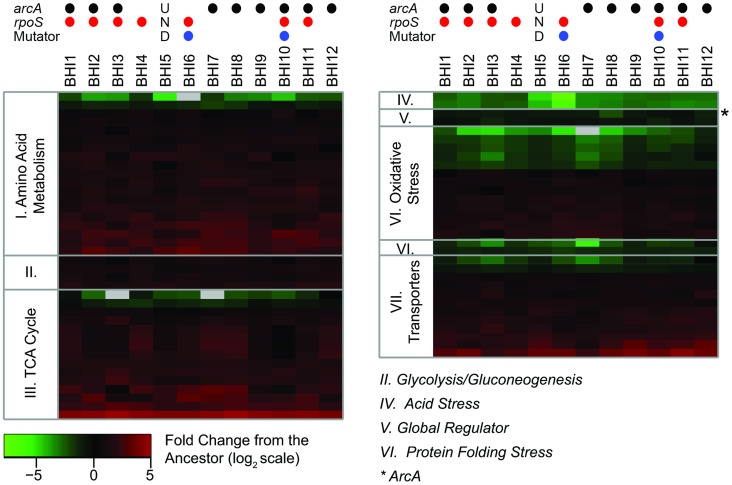
The proteomic analyses shows consistent changes across the evolved populations. We observed 488 proteins across the twelve evolved and the ancestral populations. Out of those, 166 proteins were significantly different between ancestor and evolved populations as judged by LC-MS based proteomics and then were grouped by function (S1 Text). The heat map shows the change in expression for the groups of proteins that had consistent changes with respect to the ancestor. The complete list of significantly changing proteins is given in S2 Table. Columns represent the BHI-evolved *E. coli* populations; rows represent individual proteins, grouped by functional categories. The color scale shows the average base-2 logarithm transformed fold change of each protein with respect to the ancestor sample, based on the average of triplicate measurements. Gray indicates that the protein was not detected in that population. Positive values (red) indicate higher expression in the evolved population than the ancestor; negative values (green) represent lower expression in the evolved population than the ancestor. The asterisk indicates the global regulator ArcA. The circles on the top indicate whether we identified mutations in *arcA* (black) or *rpoS* (red) in a population (at a minimum frequency of 0.05), and whether the population evolved a mutator phenotype (blue). ‘UND’ stands for undetermined as population BHI5 was excluded from the genomic analyses (see Results).

Population-level proteomic analysis of the BHI-evolved *E. coli* populations showed significant changes in protein abundance between the ancestor and evolved populations as well as a remarkable degree of parallelism among the evolved populations. We observed significant and highly parallel decreases of ArcA abundance in the evolved populations and increases of proteins of the TCA cycle, amino acid metabolism and transporters (Fig. 8). We identified 4469 unique peptides in 39 samples (ancestor and twelve evolved populations with three replicates each), corresponding to 488 proteins (see S1 Text for more details). Quantitative analysis of the 488 proteins revealed 166 proteins that were significantly different between the ancestor and the evolved populations (p<0.01; log_2_-fold change>±0.7). Of those, 58 proteins decreased and 108 proteins increased significantly over the course of the selection experiment (S2 Table). All observed proteins associated with the TCA cycle (aconitate hydratase, isocitrate dehydrogenase, 2-oxoglutarate dehydrogenase, succinyl-CoA ligase, succinate dehydrogenase, fumarate hydratase, and malate dehydrogenase) and glyoxylate shunt (isocitrate lyase) significantly increased (Fig. 8). The up-regulation of the TCA cycle and the decrease in ArcA is consistent with previous studies that observed increased flux through the TCA cycles in *arcA* knock-out mutants [50], [54]. RpoS was not detected in our proteomic analyses in any of the samples and therefore we cannot draw any conclusions about its abundance.

### Mutations in *rpoS* reduce starvation stress response and increase nutrient acquisition and utilization

Reduced starvation stress is associated with increased nutrient acquisition and metabolism and reduced stress responses [34] – conditions we expected in our resource rich environments. Altered resource utilization can be developed by increasing C/N acquisition via the up-regulation of porins, which allow nutrients to flow through the outer membrane, and a concomitant decrease of the effluxers that provide protection from toxins during starvation stress [34], [55]. Consistent with increased C/N acquisition from amino acids and small peptides, we observed significant increases of peptidases (alpha-aspartyl dipeptidase peptidase E, peptidase B, and methionine aminopeptidase) and of proteins associated with ABC transporter systems responsible for the transport of amino acids or peptides (glutamate aspartate (GltI), lysine-arginine-ornithine (ArgT), glutamine (GlnH), histidine (HisJ) and oligopeptide sytems (OppA)), and carbohydrates (galactose/methyl galactoside (MglB), ribose (RbsB), maltose/maltodextrin (MalE)).

Genomic analyses suggested some loss of function among specific efflux pumps consistent with low stress conditions. We identified mutations in several RND efflux pumps including *cmeA* and *cmeB* that are found in multiple copies within the *E. coli* and *C. freundii* genomes. Mutations in *cmeA and cmeB* ranged in frequency from 0.05 to 0.41 and occurred in 15 of 24 populations across both environments and organisms, suggesting that decreases in CmeA and CmeB function are under selection during adaptation. Eight out of twelve LB-evolved *E. coli* populations acquired mutations in either *cmeA* or *cmeB*. Mutations in *cmeA* that resulted in likely loss of function (all either insertions, deletions or SNPs to stop codons) evolved in four populations (LB5, LB9, LB11 and LB12), while five different populations had mutations in one of the *cmeB* copies (LB1, LB4, LB5, LB7 and LB8). Mutations in *cmeA and cmeB* were not as prevalent among the LB-evolved and completely absent among the BHI-evolved *C. freundii* populations. One LB-evolved *C. freundii* population acquired a substitution in *cmeB* (LB25), one had an insertion (LB34) and a third population had an insertion in the RND efflux transporter (LB2). One MA line acquired an insertion in both *cmeA* and *cmeB*. The *cmeA* and *B* mutations resulting in loss of function mutations support the SPANC balance conditions of low starvation stress and increased nutrient uptake and decreased efflux.

While *rpoS* mutants are predicted to have a decreased stress response, our proteomic data (Fig. 8, S2 Table) suggested that changes in the stress response, were more nuanced and that some stress pathways, such as the starvation and acid stress responses were up-regulated while others such as protein unfolding stress were diminished. Across the twelve BHI-evolved *E. coli* populations, we observed decreases in chaperones associated with protein folding stress (DnaK) and heat shock proteins (GroES), and in proteins involved in the oxidative stress response through glutathione (glutaredoxin 2 and 3, and glutathione peroxidase). Conversely, proteins involved in the oxidative stress response through thioredoxin (thioredoxin reductase, universal stress proteins AEFG, superoxide dismutase, glutathione S-transferase), acid stress (HdeAB) and another heat shock protein (HchA/Hsp31) increased.

### *E. coli* populations adapted to BHI show increased putrescine secretion consistent with altered nitrogen homeostasis

The up-regulation of the TCA cycle and the increased amino acid acquisition and metabolism could lead to increased production of ammonia or polyamines to maintain nitrogen homeostasis. To test this hypothesis, we determined whether the evolved populations produced and secreted more polyamines or ammonia. We began by testing the pH of spent media after 24 hours of growth, and observed a significant increase in pH from 8.1 to 8.4 in the BHI-evolved *E. coli* populations (S1 Table) compared to the ancestor. Similarly, the pH also increased significantly in the LB-evolved *C. freundii* populations, but not in the other two treatments. Increased pH is consistent with proteomic data that suggested significantly increased TCA cycle activity and amino acid metabolism. The breakdown of amino acids by the decarboxylation of ornithine or of arginine to agmatine can result in the production of the polyamine putrescine [56]. Indeed, putrescine was significantly higher in the spent media of the BHI-evolved *E. coli* population compared to the ancestor (t-test: t = 6.08, df = 22, p<0.0001), but not in the cell extract (t-test: t = 0.3, df = 20, p = 0.76)(S4 Figure). While we only have quantitative data for the BHI-evolved *E. coli* populations, the odor of the *C. freundii* populations at stationary phase suggested that they, too, all produced and secreted increased amounts of putrescine.

## Discussion

**Figure 9:**
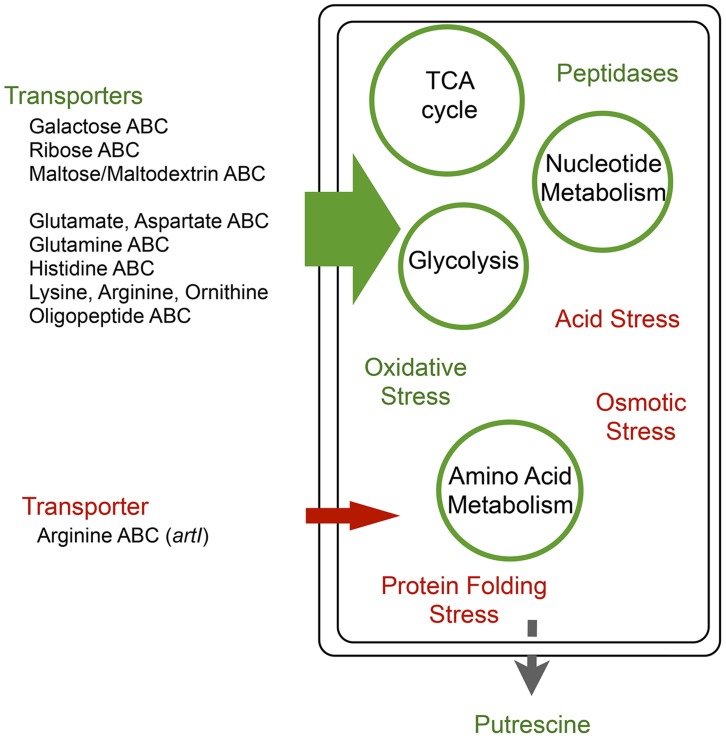
Global regulators *arcA* and *rpoS* provide a comprehensive metabolic shift during adaptation that circumvents epistatic bottlenecks. Adaptation to the amino acid rich conditions of BHI by *E.coli* are consistent with a ‘metabolic selection’ that provides a facile strategy for shifting environments. Up-regulated systems (green) are associated with the movement of abundant amino acids into the cell coupled with an increased capacity for catabolic metabolism through the TCA cycle with excess nitrogen being secreted as putrescine. Down-regulated systems (red) include components of the starvation stress response consistent with the maintenance of a new nutrient rich homeostasis.

The natural world presents organisms with complex and variable environments. Resources often range from rich and varied, to poor and limiting. An abundance of new, but usable, resources may induce very weak selection pressures and result in a complex multi-peaked adaptive landscape, where most single nucleotide changes or mutations to specific components of a metabolic pathway would not generate enough fitness gains to facilitate rapid success. One path to adaptation would be to change the global regulators that control the management of metabolic flux to provide a simple “one-step” adaptation for the entire physiology of the organism. Adaptation by such a one-step mechanism constitutes a higher order ‘metabolic selection’ that allows the organism to capture larger gains in fitness and circumvent the complications of multi-gene epistasis. To test this idea, we used wild isolates of *E. coli* and *C. freundii* and investigated their adaptive responses under weak selection as they were moved from their natural habitat, the human gut, to a rich and markedly different resource base. We found that, as expected, weak selection induced by rich complex environments resulted in large genetic variation and likely allowed even deleterious mutations to persist. We observed a striking diversity of phenotypes across all populations. Underlying genetic diversity could be observed readily as a tremendous variation in colony sizes and physical appearance on different indicator agar plates, as well as loss of motility (Fig. 2 and S1 Text). Whole population sequencing of the evolved populations identified *arcA* and *rpoS* as the targets of selection. Whole population proteomics of the BHI-evolved *E. coli* populations showed that these populations up-regulated several amino acid and carbohydrate transporters to move abundant nutrients into the cell and up-regulated the proteins of the TCA cycle needed to use them efficiently (Fig. 9). We also observed significantly increasing putrescine production consistent with increased utilization of amino acids as C/N sources. The combination of whole genome sequencing and whole population proteomics proved to be a powerful approach for the mapping of genotypic changes to biochemical mechanisms that, in turn, produce altered phenotypes.

To identify common adaptive strategies, we focused on mutations that arose repeatedly in independently evolved populations. The two most common targets of selection were *arcA* and *rpoS*, both global regulators with large pleiotropic effects. Our overall picture for adaptation is one in which the adaptation through mutations in the global regulators *arcA* and *rpoS* drive the large metabolic changes essential for adaptation to nutrient rich environments under these selection conditions. Both of these global regulators affect up to 10% of the genes within their host genome [29], [49], [50]. Mutations in *arcA* evolved consistently in the majority of the populations. ArcA consists of two domains, the receiver domain (residues 1–123) that includes the site of phosphorylation (Asp54) [57]-[59] and a DNA binding domain (124–238) [59]. Phosphorylation stimulates formation of an ArcA-P dimer that binds to a variety of specific DNA sequence motifs with high affinity to repress or activate transcription of up to 229 operons directly or indirectly in response to the environment [49], [50], [54]. The majority of the diverse mutations in *arcA* were found in the receiver domain (Fig. 7). Mapping these mutations onto the three-dimensional crystal structure (1XHE) of the receiver domain revealed that the vast majority of mutations are in surface positions and solvent accessible loops (S5 Figure), with only a few mutations mapping to the hydrophobic core. While it is likely that some of these mutations could result in a complete loss of function, the likelihood that all 46 mutations do so is slim. It is interesting that all but two mutations in *arcA* were SNPs and the two exceptions were an insertion and a deletion at the C-terminus and likely resulting in a largely functional protein. This suggests that a complete loss-of-function that eliminates *arcA* function is not as beneficial as modifying its activity and is consistent with our proteomic data that showed a decrease in ArcA levels rather than a complete absence in ArcA. Mutations within the receiver domain could decrease ArcA signaling by a number of mechanisms including: 1) reducing ArcA stability; 2) decreasing the extent of ArcA phosphorylation during signaling; 3) increasing the rate of dephosphorylation; or 4) decreasing the extent of phosphorylation-dependent oligomerization. Only a few mutations mapped to the DNA binding domain but these could also alter ArcA function by decreasing DNA binding or any of the aforementioned mechanisms for altered receiver domain function. Mutations to *arcA* were also observed in previous selection experiments, notably during adaptation to glucose limited media [60], [61] and LB [40], [62]. Interestingly, in those studies mutations in *arcA* only evolved in the aerobic cultures, suggesting that oxygen deprivation and anaerobic stress were not the driver for *arcA* mutations.

Unlike *arcA*, it was striking that all mutations to *rpoS* in the BHI-evolved *E. coli* populations and only one mutation in the other three treatments appeared to produce a complete loss of function. One explanation could be the differences in the composition of the media, mainly the presence of glucose in BHI. Carbohydrates and glucose in particular are utilized first before switching to amino acids [63]–[65]. This is reflected in a diauxic growth pattern with a second lag and exponential growth phase. The depletion of carbohydrates could induce the RpoS regulated starvation stress response, which might delay the transition to amino acid metabolism. Losing RpoS function might therefore be beneficial to a fast switching response to other nutrient sources. In LB we never observed diauxic growth patterns, which is consistent with the low concentration of carbohydrates in the media. Mutations in *rpoS* have also been shown to improve longevity during stationary phase [37]. The populations reached stationary phase within eight hours in LB and as a consequence, these populations remained in stationary phase much longer. SNPs that improve persistence in stationary phase could therefore be selected in LB.

Proteomic analyses of BHI-evolved *E. coli* populations showed increased abundance of enzymes of the TCA cycle, which is consistent with the known phenotypes of *arcA* and *rpoS* knockout mutants based on flux analyses [50], [54], [66]. Knocking out either *rpoS* or *arcA* resulted in two-fold increases in metabolic flux through the TCA cycle, while knocking out *arcB* did not have an effect on the flux through the TCA cycle, consistent with our observation that mutations in *arcB* were rare [54], [66]. While proteomics and flux analyses use different measurements, the effect of mutations in *arcA* and *rpoS* seem very similar. This is even more remarkable considering that our proteomic analyses are based on polymorphic populations and the small changes in proteomics are population averages.

In addition, we see strong up-regulation of peptidases, amino acid metabolism, amino acid transporters and oxidative stress responses, indicating that these populations are metabolizing the media at an increased rate (Fig. 8 and 9, S2 Table). Again, this pattern is consistent with previous metabolic studies [54], [66]. ArcA has been shown to either directly or indirectly regulate many operons such as amino acid and polyamine production, beta-oxidation of fatty acid and operons encoding pathways for the utilization of aromatic compounds and peptides [49]. Knock-out mutants of *rpoS* not only had increased TCA cycle activity but also increased amino acid metabolism [66].

The arginine, asparagine and glutamine metabolism pathways and the TCA cycle feed into the urea cycle. While we did not observe changes in expression of enzymes of the urea cycle, we observed a significant increase in the production and secretion of putrescine in BHI-adapted population. The polyamine putrescine can be produced during the breakdown of amino acids by the decarboxylation of ornithine or by the decarboxylation of arginine via agmatine [56]. Arginine decarboxylase has been proposed to localize in the cell envelope, where it converts exogenous arginine to putrescine via agmatine [67]. Because we observed a significant decrease of agmatinase in the evolved populations, it seems more likely that putrescine is produced from ornithine, a component of the urea cycle. In the adapted populations, putrescine might be acting as a nitrogen sink for catabolism of amino acids via the urea cycle or, alternatively, may help with the increased oxidative stress resulting from increased metabolism. There are many roles for polyamines in metabolism including as C/N sources, oxidative stress response, and signaling [68]–[73], so an understanding of increased secretion of putrescine by our adaptive populations will require further biochemical studies.

We expected that parallel evolution could also evolve along pathways and lead to phenotypic convergence by mutating different genes along the pathways [12], [15]. Indeed, we did see some convergence in the regulation of *rpoS*, where not all populations had mutations in *rpoS* and instead had mutations in genes that regulate the expression of *rpoS*. We interrogated the genomic data for such phenotypic convergence by analyzing parallel evolution for different functional categories, but did not observe any evidence for phenotypic convergence at different levels of increasing complexity (see S1 Text, S6–S8 Figure). Nonetheless, we did see a high degree of parallel evolution among the populations when we grouped the proteins with significant changes to pathways. This is even more remarkable considering that we performed our proteomic analyses on whole, polymorphic populations and as such only measure the average change of a populations compared to the ancestor. This parallelism shows a clear response to selection. The global up-regulation of metabolism and the lack of clear phenotypic convergence of mutations along the pathway further support our assertion that mutations in *arcA* and *rpoS* drive adaptation to the rich selective conditions and lead to the observed metabolic changes. These relatively small changes observed in the proteomic data also further support our previous observations that very small biochemical changes can have large fitness effects [74] and are likely very relevant to adaptation in nature.

The consistent evolution of mutations in global regulators *arcA* and *rpoS* that each affect expression of about 10% of the genome supports the model in which adaptation evolved through the evolution of a few mutations with large beneficial effects. Instead of acquiring beneficial mutations in every gene involved in the TCA cycle and the various amino acid metabolism pathways, acquiring mutations in regulators affecting all these genes simultaneous is undoubtedly more efficient. Selection studies in other, less complex environments also implicated mutations in global regulators that lead to stable coexistence and polymorphic populations suggesting that mutations in global regulators are beneficial in different environments [60], [61]. In contrast to those earlier studies under glucose-limiting conditions, mutations to *arcA* and *rpoS* arose very rapidly and repeatedly across our populations. Diverse phenotypes and genotypes suggest that our populations are polymorphic as well. For example, the sequential utilization of carbohydrates and amino acids [64], [65] could select for different mutations specialized to either carbohydrate or amino acid utilizations, similar to the stable coexistence of different temporal specialist observed in previous studies [10], [19], [20], [25]. It is possible that *arcA* and *rpoS* mutations provide selective benefits in different phases of the growth cycle and the coexistence of these mutations in the populations could be an indication of such temporal and potentially nutritional specialization.

By investigating adaptation of wild organisms to resource rich environments we have shown that adaptation occurs within five hundred generations through mutations in global regulators, leading to increased rates of metabolism. Mutations to global regulators might be more common during selection in permissive environments. At niche boundaries such as a thermal limits, single mutations could greatly increase the fitness of an organism [75], mostly because fitness at the niche boundaries can be dramatically reduced compared to the niche optimum [76], [77] and thus a small number of mutations or even a single mutation can result in substantial fitness gains.

Both *rpoS* and *arcA* have been linked to virulence [78], [79]. Our findings suggest that it is important to appreciate the role of laboratory adaptation when evaluating strains for pathogenicity, especially in light of the fact that *rpoS* loss-of-function mutations evolve readily in the laboratory but are not found in natural populations [38]. The transition from the natural habitat to laboratory conditions suggests how we might improve experimental evolution studies that require handling and adaptation of pathogens as well as provide a starting point for forensic attribution of strains during outbreaks of novel pathogens.

## Materials and Methods

### Strain isolation and identification

*E.coli* RU1 (hence forth referred to as *E. coli*) and *C. freundii* RU2 (hence forth referred to as *C. freundii*) were isolated from the stool of healthy humans (S1 Text, S5 Figure). To minimize any potential for adaptation during the isolation process, we plated stool samples on MacConkey agar plates. After a single overnight growth, half of a single colony was flash frozen at −80°C in Trypticase soy broth with 20% Glycerol (BD, USA) and the other half was used for phenotypic strain characterization. Strain identification by 16S sequencing was done from the frozen sample. All experimental evolution studies started from the frozen primary isolates by using a single clonalized colony derived from the initial snap frozen isolate. The identity of the wild "un-adapted" strains was based on the results of API 20 E (Biomerieux, USA) test strips and species-specific PCR (using forward primer: AGAGTTTGATCMTGGCTCAG, reverse primer: GWATTACCGCGGCKGCTG).

### Selection experiments

#### Single colony transfers (mutation accumulation control experiments)

Twelve clones of *E. coli* RU1 were evolved independently for 200 days by daily transfers of a single, randomly chosen colony to a fresh LB plate (Fig. 1). Every 15 days, a sample of the transferred colony was frozen at −80°C. To calculate the generations per transfer, we assessed the number of cells in a colony after 24 hours of growth on LB agar and calculated the number of doublings as log_2_(number of cells/colony) at generation 0, 105 and 200 for two randomly chosen lines. The average number of generations per transfer was calculated as 29.4, which results in 5888 generations over 200 single cell bottlenecks.

#### Population flask transfers (experimental evolution of populations)

A single colony from the frozen cultures of *E. coli* RU1 and C. *freundii* RU2 were each used to inoculate twelve independent cultures in Luria Broth Miller (LB, BD) and brain-heart infusion (BBL BHI, BD) (Fig. 1, S3 Table). Populations evolved in liquid media were transferred as described previously [10], [11]. LB-evolved populations were grown in 10 ml liquid LB Miller broth in 25mm test tubes at 250 rpm and 37°C, and transferred daily by 100-fold dilutions into fresh media (∼6.6 generations/day) for a total of 75 days or 500 generations. Every 15 days, we froze a sample of the populations at −80°C for further analyses. The BHI-evolved populations were cultured in 10 ml of BHI broth under the same conditions as the LB cultures, but transferred daily by 40,000-fold dilutions into fresh media (∼15.2 generation/day) for a total of 50 days or 765 generations. This dilution was chosen to achieve the same effective population size at transfer of 10^5^ cells/ml as in a parallel experiment performed with *Yersinia pestis* to allow direct comparisons. The BHI-evolved populations were frozen at −80°C after 25 and 50 days.

### Phenotypic assays

Assays were performed either at the population level or the single colony level. Cells or populations were grown in their selective media (LB or BHI) and grown in liquid media or plated on agar plates made with their selective media, unless otherwise stated. Adaptation to the selective environments was assessed as changes in lag time and growth rate by measuring OD_600_ over 24 hours of growth in liquid media following Walkiewicz *et al.* [74]. To test for changes in the pH of spent media, we grew the populations to stationary phase and measured the pH of the media after removing the cells. To test for genetic variation within the populations, we plated the populations at low density on tetrazolium arabinose plates and observed considerable variation in both colony size and in the ability to utilize arabinose. We plated the populations on the selective media supplemented with agar and isolated eight randomly chosen colonies from each of the 12 populations per treatment. These test sets of 96 individual isolates per species and environment were used for three phenotypic assays: 1) the redox state by plating on methylene blue (0.065 g/liter); 2) differences in exopolysaccharides by plating on Congo Red (0.15 g/liter); and 3) loss of motility by plating cells on soft agar (0.25% DIFCO). For more information see S1 Text.

### Sequencing, alignment and mutation identification

#### Ancestral genomes

Genomic DNA of the two ancestral strains was isolated using the Ultra Clean Microbial DNA isolation Kit (Mo Bio Laboratories, Inc.) and sequenced on the Roche 454 platform according to standard sequencing methods. The reference sequences of *E. coli* RU1 and *C. freundii* RU2 were assembled *de novo* using Newbler v2.6 and annotated using RAST [80]. The annotated genes were grouped by function and assigned to subsystems using SEED [81] (S6 Figure).

#### Evolved lines and whole populations sequencing

Genomic DNA of single clones (MA lines: MA1–MA12) and whole populations (LB1–LB12, LB25–LB36, and BHI1–BHI24) was isolated as described for the ancestors and sequenced on Illumina HiSeq in 100 bp paired-end reads. Reads were aligned to the reference sequence using the breseq-0.24.rc6 pipeline (with options –j2 –c –p)[82]. Mutations that occurred at a minimum frequency of 0.05, which is an order of magnitude above the overall error rate as determined following Saxer et al. [44] were considered. Because we used such a low acceptance threshold for mutations in polymorphic populations, we used additional measures to reduce the false positive rate, that can be due to errors in the reference sequence, duplications that were incorrectly resolved in the reference sequence, repeat regions or sequence properties, or methylation patterns [83]. Mutations arise at random, and under a model of selective neutrality, the frequency of a mutation should be proportional to the time it arose, while the frequency of a beneficial mutation should be determined by the time it arose and by its selection coefficient. Therefore, mutations that arise at random cannot be expected to occur at almost the same frequency in replicated polymorphic populations. In clonal samples, polymorphic sites could arise when a single colony is grown to high density for DNA isolation. However, such mutations should be rare within a single sample and very unlikely to occur in multiple independent clonal samples. In addition, the relaxed selection experience by the MA lines makes it unlikely that the same mutation would arise in multiple independently evolved lines. While beneficial mutations can evolve more than once in mutation accumulation experiments, it is unlikely that such mutations would occur at the exact same site (nucleotide) at approximately the same frequencies in independently evolved mutation accumulation lines, and that these same mutations would be polymorphic in a clonal sample (i.e. at lower frequency than 0.8–0.9 among reads aligning to that site).

We aligned the reads of the clonal MA lines as if they were polymorphic populations and identified mutations that occurred in 4 or more lines. These sites were removed from BHI- and LB-evolved populations. We performed the same removal procedure for mutations that occurred in one to twelve MA lines and observed a significant change in the number of mutations removed based on their presence in three or four MA lines. This resulted in the removal of 233 mutations that occurred in three treatments (LB, BHI and MA), 72 mutations that occurred in two treatments (MA and LB, or MA and BHI) and 3 mutations that occurred in the MA lines. The majority of these mutations were in intergenic regions, in transposable elements, hypothetical genes and in the large and small ribosomal subunits. In addition, we removed mutations in genes encoding for genes annotated as core proteins, the Magnesium and cobalt transport protein, CorA and tRNA dihydrouridine synthase A.

Based on our experience with *E. coli* and the work of Lang et al. [84], we used a different approach to filter out false positive mutations for *C. freundii*. Adaptive mutations that arise in multiple populations evolve at different times and in populations with a different assortment of genotypes, which is reflected in different frequencies in the populations. The frequencies of false positive mutations that are a result of systematic errors on the other hand will be very similar among populations. Therefore, we filtered out any site for which the frequencies in BHI and LB did not significantly differ based on Levene's test [85].

### Proteomic and putrescine analyses of evolved populations

Cell extracts and spent media samples were prepared by growing the evolved populations and ancestral populations to stationary phase, separating the cells from the supernatant by centrifugation and inactivating remaining cells with 70% (v/v) ethyl alcohol.

#### LC-MS analysis of proteins

Protein was extracted by re-suspending cell pellets in 50 µL lysis buffer [6 M urea (Sigma U-0631) and 14.3 mM 2-mercaptoethanol (Sigma M6240) in 100 mM triethylammonium bicarbonate (TEAB), pH 9 (Sigma T7408)]. The extracted protein was digested with trypsin and the tryptic peptides analyzed by LC-MS using the accurate mass and time (AMT) tag proteomics approach [86]. See S1 Text for details. The software program DAnTE [87] was employed to perform an abundance roll-up procedure to convert peptide abundance information to protein information, thereby inferring protein abundances. ANOVA analyses were applied to protein abundance data sets (*p*-value ≤0.01) to identify statistically significant differences in protein expression levels (S2 Table, S9 Figure).

#### Putrescine secretion

Putrescine concentration in the cell extract and in spent media was measured during GC-MS analyses of carbohydrates. For specific details see S1 Text.

## Supporting Information

S1 Figure *E. coli* RU1 and *C. freundii* RU2 are closely related but group with members of their own species. Phylogenetic analyses of the two ancestral species show that *E. coli* RU1 (A) groups with non-pathogenic *E. coli* strains (indicated by the green bar; red bar indicates pathogenic strains), and *C. freundii* RU2 groups with other *C. freundii* (b) strains (neighbor joining tree).
(TIF)

S2 Figure Phenotypic variation in redox state and exopolysaccharide content was evident both within and among populations when plated on methylene blue plates (A, B) or Congo Red plates (C, D), respectively. Eight single colonies of twelve *E. coli* populations (one population per column) evolved in LB (A, C) and BHI (B, D) on LB agar plates supplemented with methylene blue (A, B) and Congo Red (C, D) with the ancestral strain plated eight times at the bottom of the plate for comparisons.
(TIF)

S3 Figure Mutations in *rpoS* include predominantly stop codons, large deletions and frame shifts. Among the LB-evolved *E. coli* (A) and *C. freundii* populations (B) and the BHI-evolved *C. freundii* populations (D) SNPs were more common, while mutations to stop codons or frame shift dominated in the BHI-evolved *E. coli* populations (C). The evolved mutations are quite diverse, but less so than was observed for *arcA*. The blue bar represents the gene and the white lines the location of the mutations. The actual change is indicated. The star represents a mutation to a stop codon. Deletions are denoted as minus the number of deleted nucleotides (e.g. -4nt). Every deletion resulted in a frame shift. Red dots indicate the number of populations with a specific mutation.
(TIF)

S4 Figure Putrescine did not change in the cell extract (A), but increased significantly in the spent media (B). Ribose, mannose and glucose content in the cell extract changed significantly over the course of the selection experiment (mean of twelve ancestral (blue) or twelve BHI-evolved (red) populations and 95% CI). Star indicates significance after sequential Bonferroni correction.
(TIF)

S5 Figure Mutations to *arcA* were mapped onto the three dimensional structure of *E. coli* ArcA receiver domain (1XHE). The large majority of mutations mapped onto surface accessible positions (blue) consistent with attenuation of ArcA function but not a total loss of function. A small number of mutations (red) did map into locations with the nonpolar core and are more likely to cause a loss of function. None of the mutations introduced stop codons and only two introduced small deletions in the DNA binding domain (not shown) at the C-terminus of the protein.
(TIF)

S6 Figure The subsystem category distributions are very similar for *E. coli* (A) and *C. freundii* (B). A slightly larger percent of the genes are assigned to subsystem categories for *E. coli* (61%) than for *C. freundii* (59%) with a larger percent of genes assigned to the iron acquisition and metabolism category in *C. freundii* than in *E. coli*.
(TIF)

S7 Figure Parallel evolution at the level of categories is relatively rare. The number of mutations in a particular category is plotted of the number of populations with mutations in that category. Most categories acquired mutations in only a few populations. Nonetheless, we observe parallel evolution at the gene level with mutations evolving in all populations evolved in the same media. The degree of parallelism decreases when we consider mutations at the subsystem level. At the level of categories, we see an increased level of parallelism again. The LB-evolved *C. freundii* populations show the highest degree of parallel evolution across all levels. The dots indicate all categories with mutations. A select number of categories with parallel responses are highlighted.
(TIF)

S8 Figure The number of genes in different subsystem categories varied considerably (A). Adjusting for the number of genes in each category using the subsystem scores (see S1 Text for more information), we did not observe a consistent response to the selective environment in terms of mutations that evolved in each category in LB (B) or BHI (C). The numbers of genes in each subsystem category are plotted for *E. coli* (green) and *C. freundii* (blue) based on the annotations in RAST and SEED. Fig. B and C show the subsystem scores minus one for every subsystem for *E. coli* (green) and *C. freundii* (blue) evolved in LB (B) and in BHI(C). The subsystem score is calculated as the ration of the number of genes with mutations in a subsystem divided by the number of total genes in that subsystem and the total number of genes with mutations divided by the total number of genes (for that species). As such, it standardizes the number of genes with mutations per subsystems and per species to allow direct comparisons.
(TIF)

S9 Figure *E. coli* ancestor and evolved populations show marked differences in protein abundance profile inferred from measured peptides. (A) Principal components analysis of LC-MS identified peptides and their abundances. Similarities and differences in peptide abundances due to laboratory evolution drove the clustering and segregation of data within and across treatment, respectively. The inset shows the variability captured by each principal component. Red dots labeled as T0 in the legend represent triplicate analyses of the ancestor strain. Blue dots labeled as T50 in the legend represent triplicate analyses of twelve lineages of the evolved strain. (B) Heat map of Pearson correlation coefficients of LC-MS derived peptide abundance profiles. Each row or column represents an LC-MS analysis, and each cell represents the Pearson correlation coefficient between the peptide abundance vectors for each binary comparison. The color of each cell represents the value of the Pearson correlation coefficient for each binary comparison. The “Color Key” plot denotes the scale of the Pearson correlation coefficient and is approximately 0.7–1, indicating highly similar and highly distinct peptide abundance profiles across the samples analyzed. Color bars indicate sample groups: T0, triplicate analysis of ancestor strain; T50, triplicate analysis of 12 lineages of evolved strain. Pearson correlation plot of measured peptide abundances across the ancestor and evolved population samples.
(TIF)

S1 Table Phenotypic changes in lag time, growth rate, stationary phase density and pH of spent media over the course of the selection experiment. The F and p-values are based on planned comparisons in a single factor ANOVA comparing the ancestor to the evolved populations, which were assayed as whole, polymorphic populations.
(DOCX)

S2 Table Proteins that changed significantly over the course of adaptation. The table lists the log2 changes from the average of the ancestor for three replicates for the ancestor and all the evolved populations for every protein that changed significantly. The proteins are annotated into functional classes. The heat map in Fig. 8 is based of a select functional classes.
(XLSX)

S3 Table Estimated media composition based on information provided in BD Diagnostics in Difco & BBL Manual – of Microbiological Culture Media and in BD Bionutrients Technical Manual – Advance Bioprocessing. The composition of individual media components (e.g. tryptone) were listed for the individual components of the media (e.g. tryptone) as % of the dry weight (e.g. total glutamic acid in tryptone is 15%). To estimate the final concentration of the different amino acids in the media, we calculated the content in each component (e.g. tryptone) and accounted for how much of this component was used in the media. The final % for the individual ingredients were summed up over all the components of the media. Note: BHI estimates to not contain “brain heart infusion from solids”.
(DOCX)

S1 Text Detailed information on the ancestral genomes, phenotypic assays of evolved populations, mutator genotypes, evolution of *arcA* in populations of a laboratory adapted strain, carbohydrate analyses, subsystem analyses, proteomic analyses and methods.
(PDF)
