# Supplementary figures and images for: Mutations in Global Regulators Lead to Metabolic Selection during Adaptation to Complex Environments
Source: PLoS Genet. 2014 Dec 11;10(12):e1004872. doi: 10.1371/journal.pgen.1004872 (PMC4263409; doi:10.1371/journal.pgen.1004872)

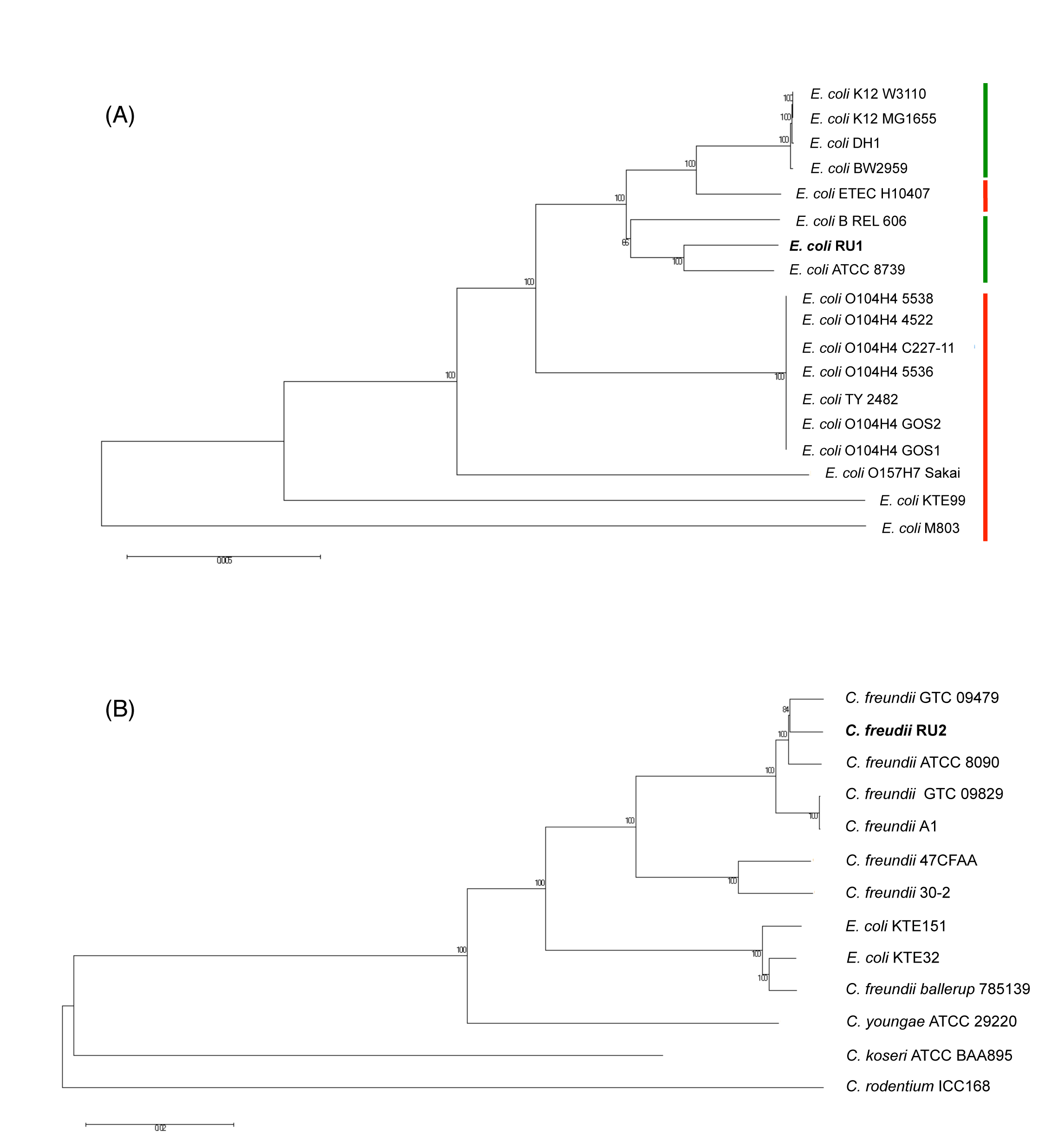

Supplement: S1 Figure — E. coli RU1 and C. freundii RU2 are closely related but group with members of their own species. Phylogenetic analyses of the two ancestral species show that E. coli RU1 (A) groups with non-pathogenic E. coli strains (indicated by the green bar; red bar indicates pathogenic strains), and C. freundii RU2 groups with other C. freundii (b) strains (neighbor joining tree). (TIF) [file pgen.1004872.s001.tif]

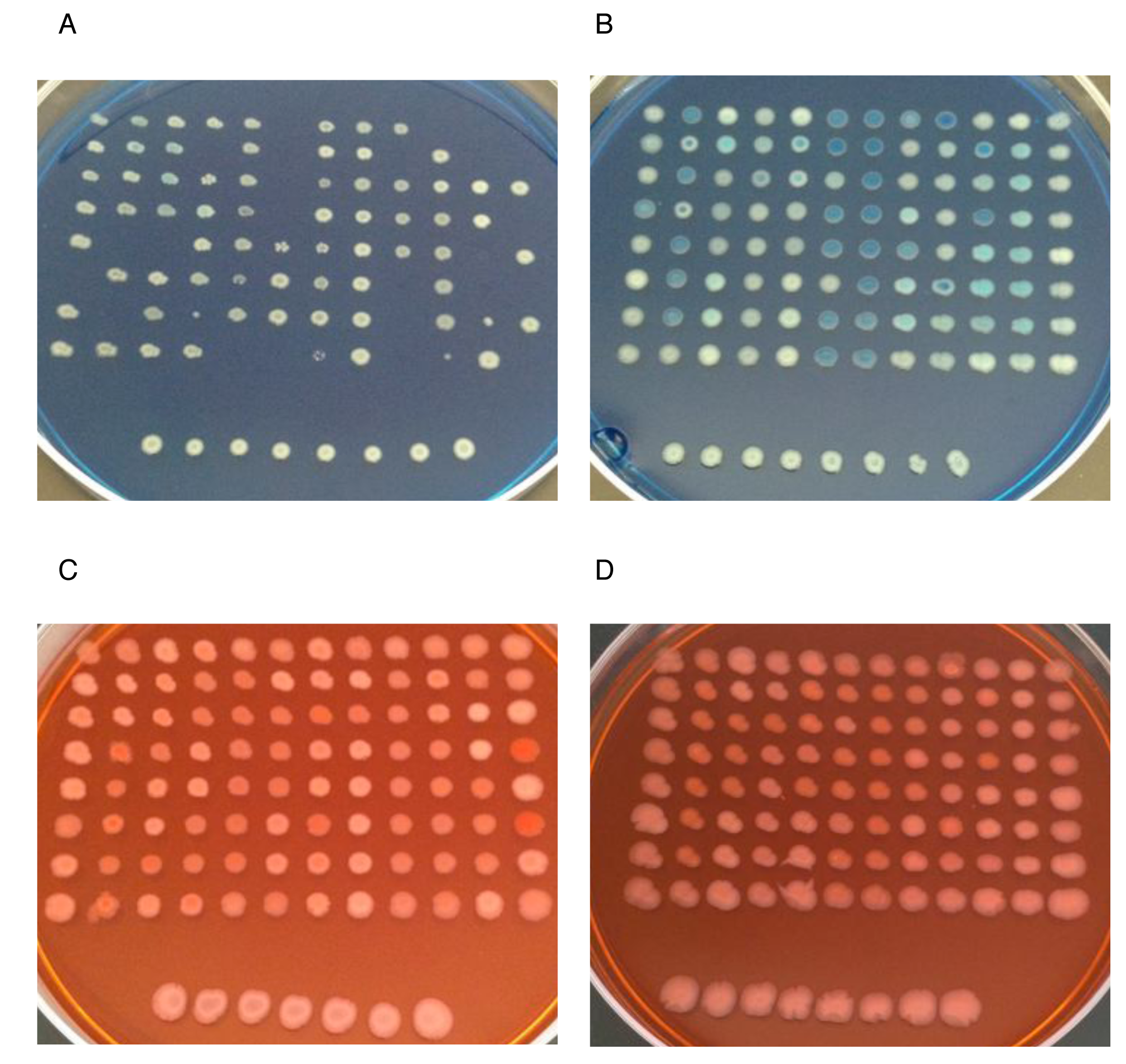

Supplement: S2 Figure — Phenotypic variation in redox state and exopolysaccharide content was evident both within and among populations when plated on methylene blue plates (A, B) or Congo Red plates (C, D), respectively. Eight single colonies of twelve E. coli populations (one population per column) evolved in LB (A, C) and BHI (B, D) on LB agar plates supplemented with methylene blue (A, B) and Congo Red (C, D) with the ancestral strain plated eight times at the bottom of the plate for comparisons. (TIF) [file pgen.1004872.s002.tif]

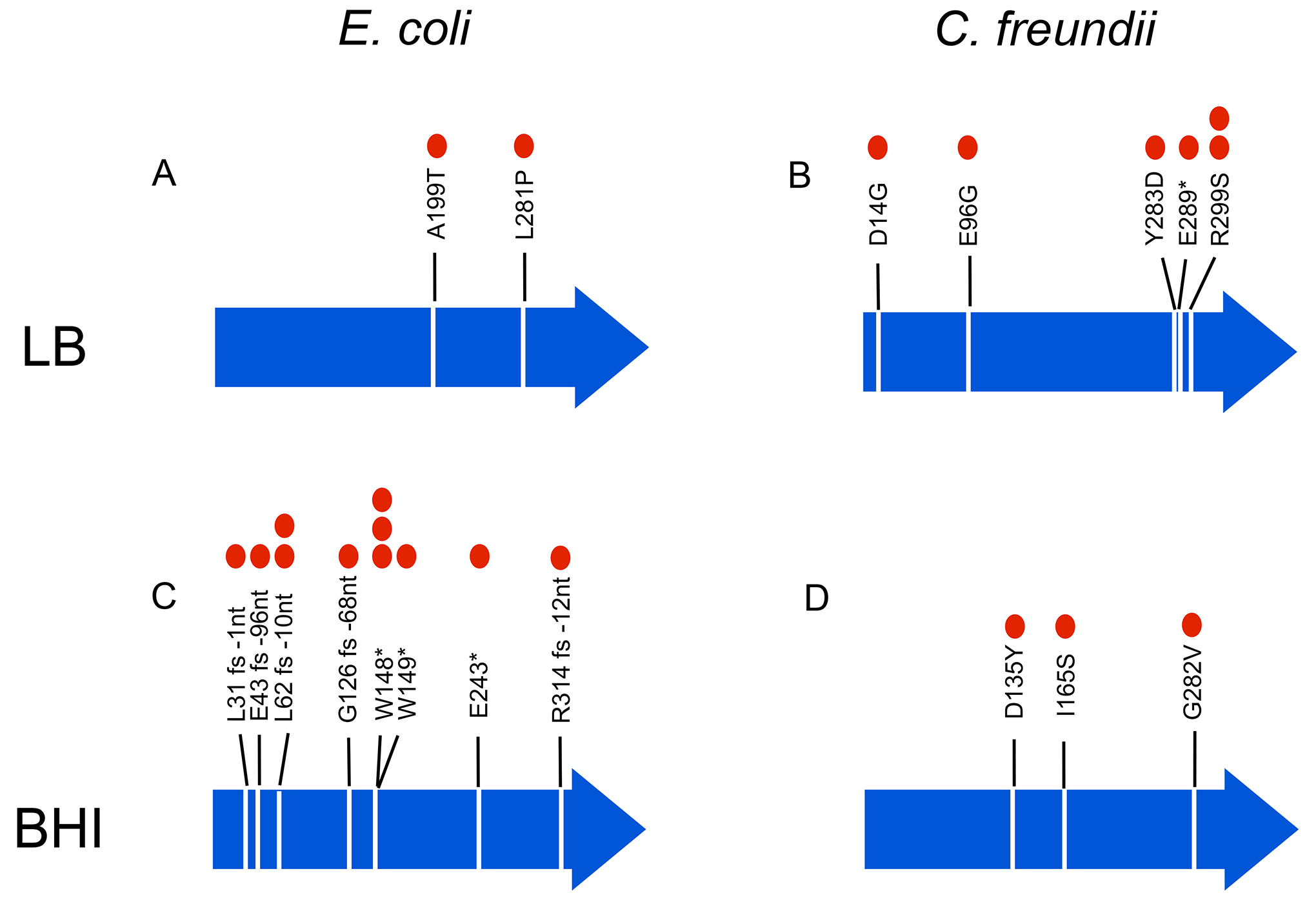

Supplement: S3 Figure — Mutations in rpoS include predominantly stop codons, large deletions and frame shifts. Among the LB-evolved E. coli (A) and C. freundii populations (B) and the BHI-evolved C. freundii populations (D) SNPs were more common, while mutations to stop codons or frame shift dominated in the BHI-evolved E. coli populations (C). The evolved mutations are quite diverse, but less so than was observed for arcA. The blue bar represents the gene and the white lines the location of the mutations. The actual change is indicated. The star represents a mutation to a stop codon. Deletions are denoted as minus the number of deleted nucleotides (e.g. -4nt). Every deletion resulted in a frame shift. Red dots indicate the number of populations with a specific mutation. (TIF) [file pgen.1004872.s003.tif]

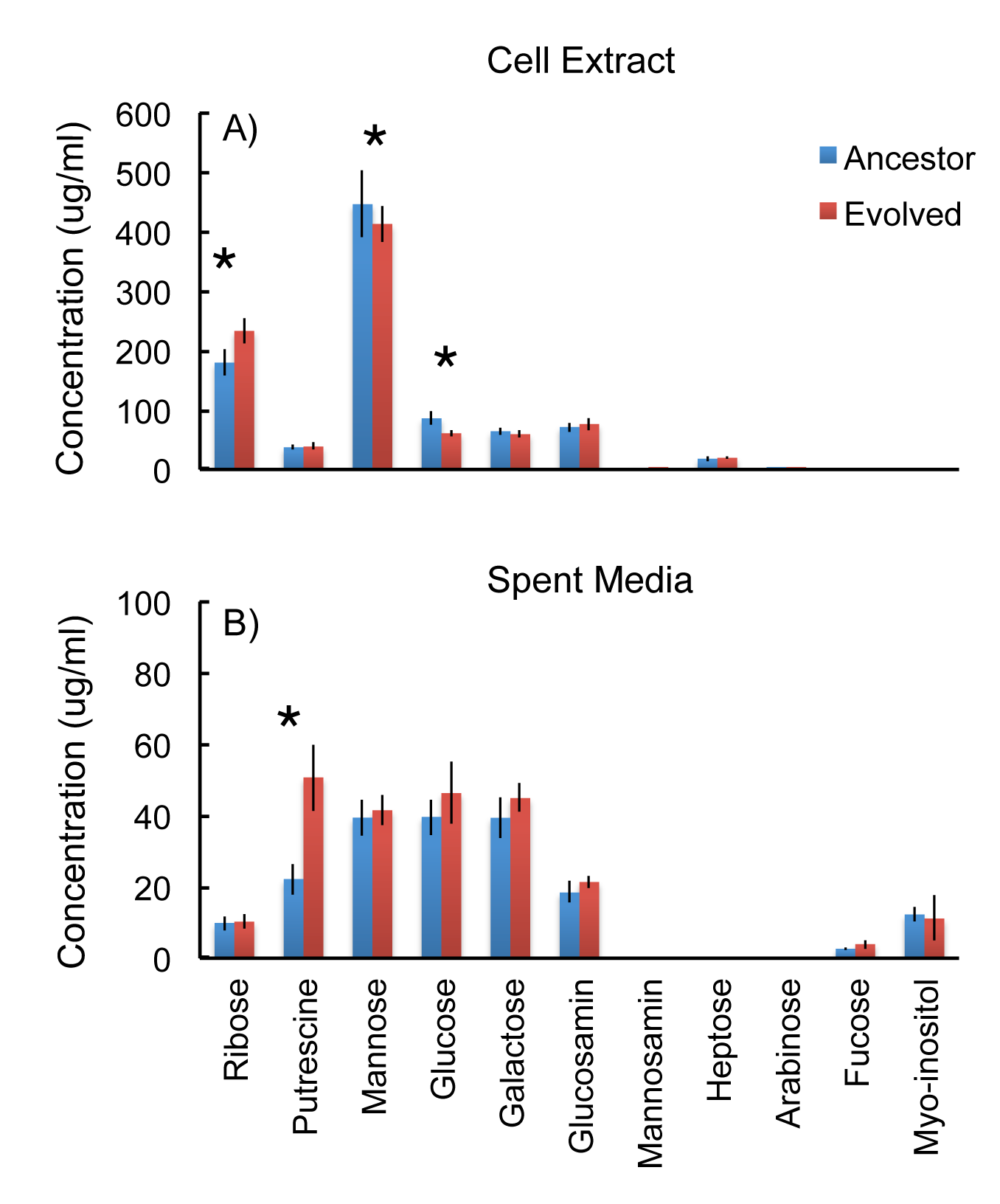

Supplement: S4 Figure — Putrescine did not change in the cell extract (A), but increased significantly in the spent media (B). Ribose, mannose and glucose content in the cell extract changed significantly over the course of the selection experiment (mean of twelve ancestral (blue) or twelve BHI-evolved (red) populations and 95% CI). Star indicates significance after sequential Bonferroni correction. (TIF) [file pgen.1004872.s004.tif]

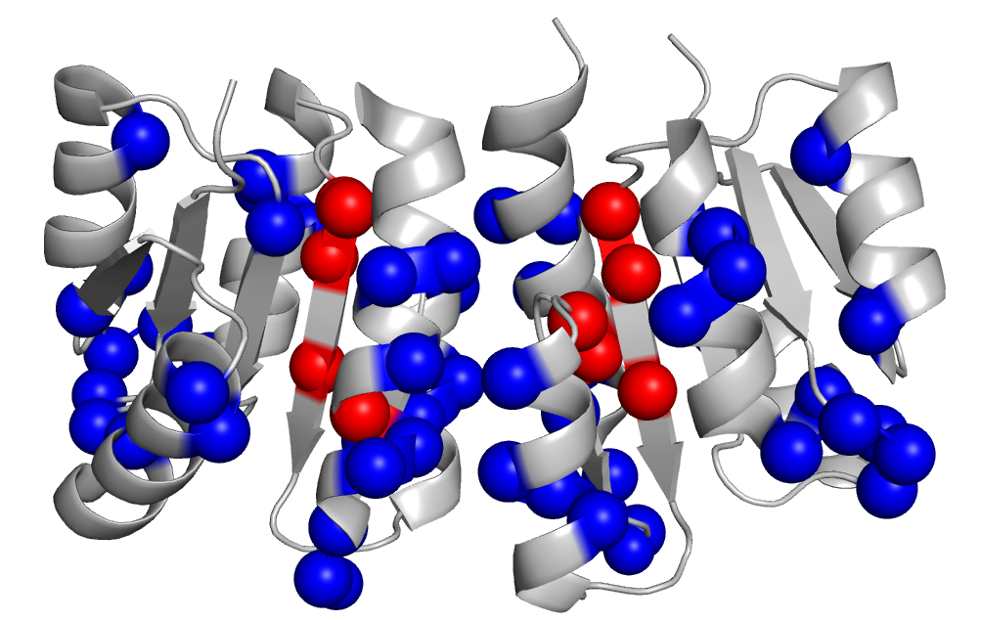

Supplement: S5 Figure — Mutations to arcA were mapped onto the three dimensional structure of E. coli ArcA receiver domain (1XHE). The large majority of mutations mapped onto surface accessible positions (blue) consistent with attenuation of ArcA function but not a total loss of function. A small number of mutations (red) did map into locations with the nonpolar core and are more likely to cause a loss of function. None of the mutations introduced stop codons and only two introduced small deletions in the DNA binding domain (not shown) at the C-terminus of the protein. (TIF) [file pgen.1004872.s005.tif]

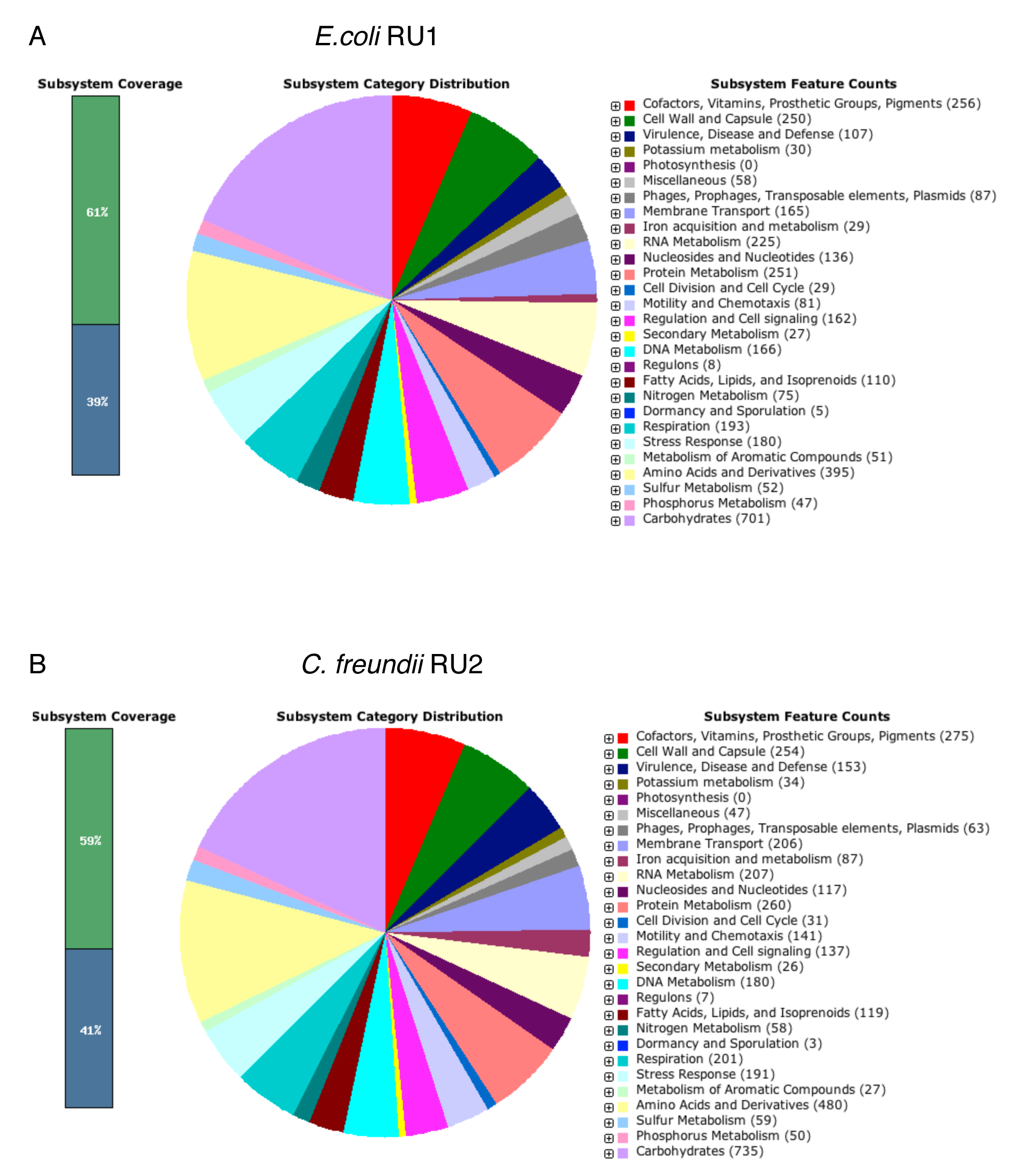

Supplement: S6 Figure — The subsystem category distributions are very similar for E. coli (A) and C. freundii (B). A slightly larger percent of the genes are assigned to subsystem categories for E. coli (61%) than for C. freundii (59%) with a larger percent of genes assigned to the iron acquisition and metabolism category in C. freundii than in E. coli. (TIF) [file pgen.1004872.s006.tif]

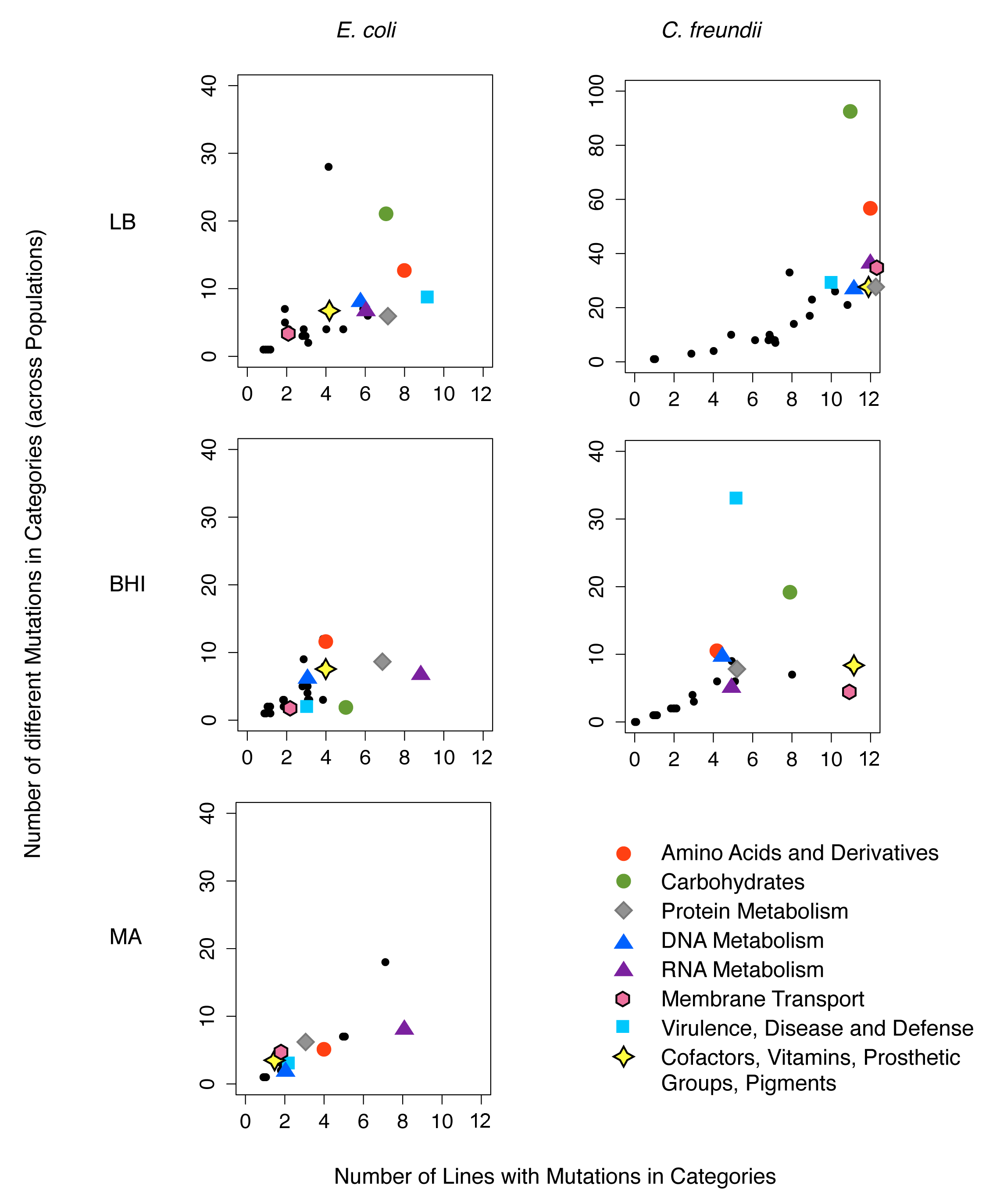

Supplement: S7 Figure — Parallel evolution at the level of categories is relatively rare. The number of mutations in a particular category is plotted of the number of populations with mutations in that category. Most categories acquired mutations in only a few populations. Nonetheless, we observe parallel evolution at the gene level with mutations evolving in all populations evolved in the same media. The degree of parallelism decreases when we consider mutations at the subsystem level. At the level of categories, we see an increased level of parallelism again. The LB-evolved C. freundii populations show the highest degree of parallel evolution across all levels. The dots indicate all categories with mutations. A select number of categories with parallel responses are highlighted. (TIF) [file pgen.1004872.s007.tif]

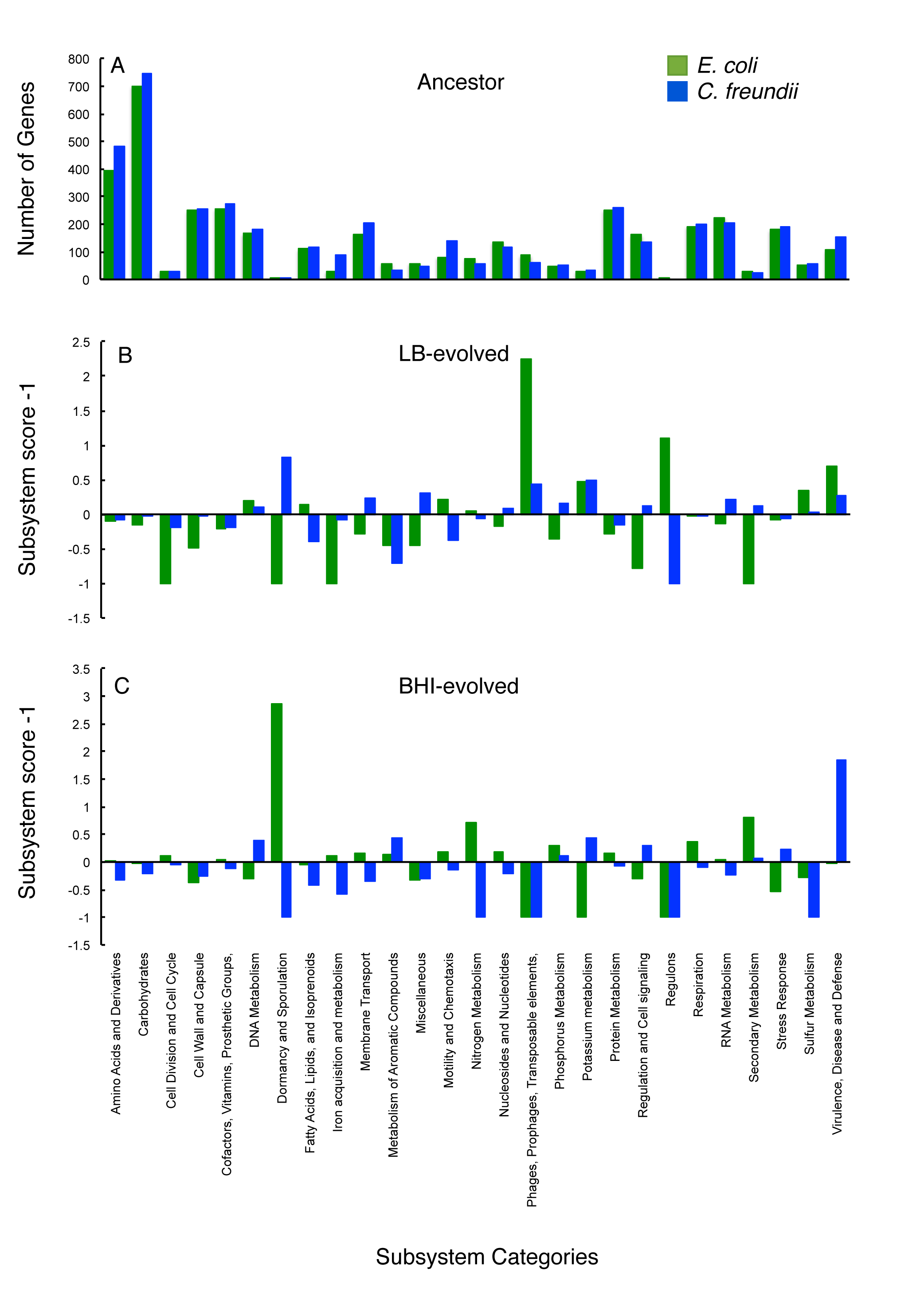

Supplement: S8 Figure — The number of genes in different subsystem categories varied considerably (A). Adjusting for the number of genes in each category using the subsystem scores (see S1 Text for more information), we did not observe a consistent response to the selective environment in terms of mutations that evolved in each category in LB (B) or BHI (C). The numbers of genes in each subsystem category are plotted for E. coli (green) and C. freundii (blue) based on the annotations in RAST and SEED. Fig. B and C show the subsystem scores minus one for every subsystem for E. coli (green) and C. freundii (blue) evolved in LB (B) and in BHI(C). The subsystem score is calculated as the ration of the number of genes with mutations in a subsystem divided by the number of total genes in that subsystem and the total number of genes with mutations divided by the total number of genes (for that species). As such, it standardizes the number of genes with mutations per subsystems and per species to allow direct comparisons. (TIF) [file pgen.1004872.s008.tif]

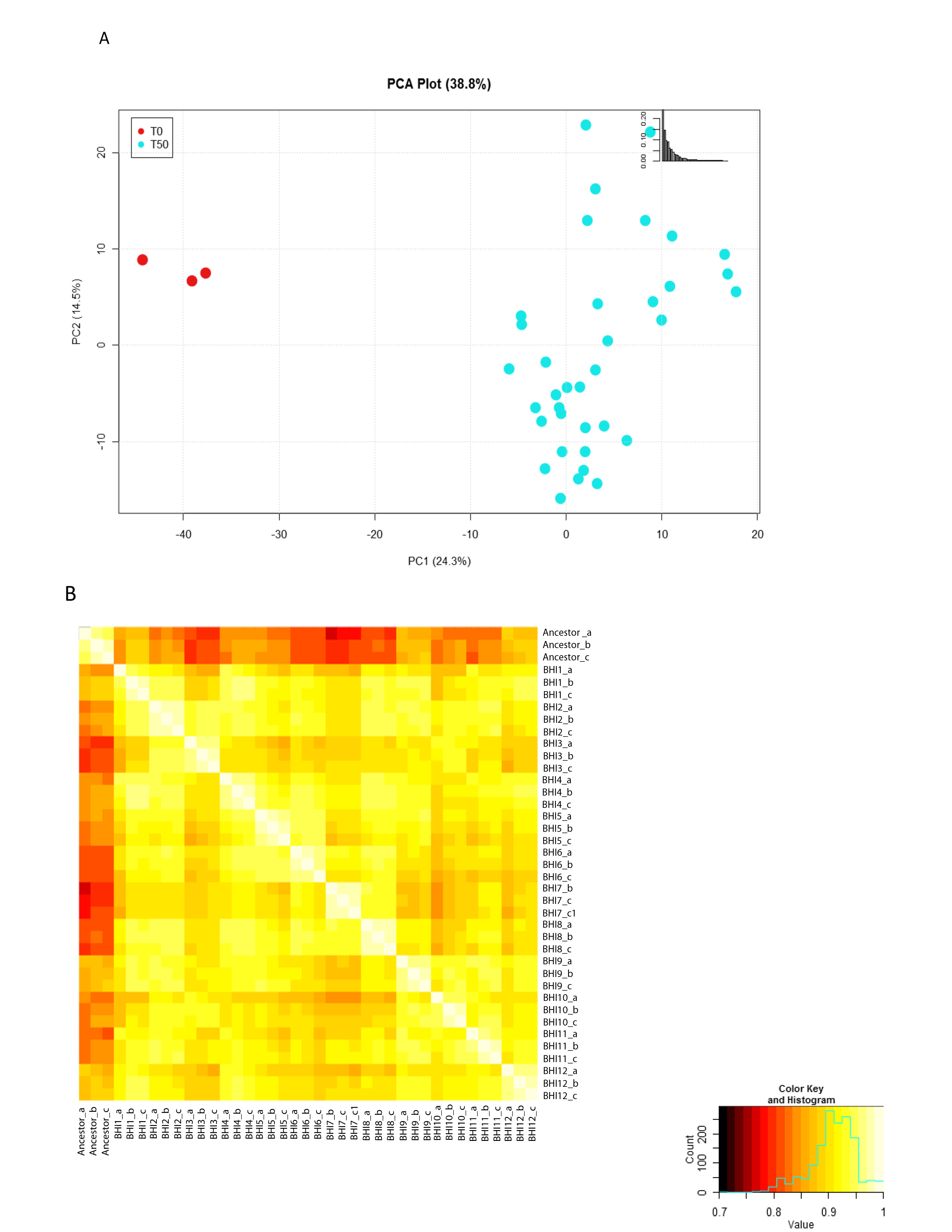

Supplement: S9 Figure — E. coli ancestor and evolved populations show marked differences in protein abundance profile inferred from measured peptides. (A) Principal components analysis of LC-MS identified peptides and their abundances. Similarities and differences in peptide abundances due to laboratory evolution drove the clustering and segregation of data within and across treatment, respectively. The inset shows the variability captured by each principal component. Red dots labeled as T0 in the legend represent triplicate analyses of the ancestor strain. Blue dots labeled as T50 in the legend represent triplicate analyses of twelve lineages of the evolved strain. (B) Heat map of Pearson correlation coefficients of LC-MS derived peptide abundance profiles. Each row or column represents an LC-MS analysis, and each cell represents the Pearson correlation coefficient between the peptide abundance vectors for each binary comparison. The color of each cell represents the value of the Pearson correlation coefficient for each binary comparison. The “Color Key” plot denotes the scale of the Pearson correlation coefficient and is approximately 0.7–1, indicating highly similar and highly distinct peptide abundance profiles across the samples analyzed. Color bars indicate sample groups: T0, triplicate analysis of ancestor strain; T50, triplicate analysis of 12 lineages of evolved strain. Pearson correlation plot of measured peptide abundances across the ancestor and evolved population samples. (TIF) [file pgen.1004872.s009.tif]
