# Supplementary material for: Mutations in Global Regulators Lead to Metabolic Selection during Adaptation to Complex Environments
Source: PLoS Genet. 2014 Dec 11;10(12):e1004872. doi: 10.1371/journal.pgen.1004872 (PMC4263409; doi:10.1371/journal.pgen.1004872)
Supplement: S1 Table — Phenotypic changes in lag time, growth rate, stationary phase density and pH of spent media over the course of the selection experiment. The F and p-values are based on planned comparisons in a single factor ANOVA comparing the ancestor to the evolved populations, which were assayed as whole, polymorphic populations. (DOCX) [file pgen.1004872.s010.docx]

| Table S1: Phenotypic changes in growth rate, lag time, stationary phase density and pH of spent media | | | | | | | | | | |
| --- | --- | --- | --- | --- | --- | --- | --- | --- | --- | --- |
| Trait | Media | Species | Ancestor | Evolved | |  |  |  |  |  |
|  |  |  | Mean | Mean | Df^1^ | SS^2^ | F^3^ | p^4^ |  |  |
| Maximum Growth Rate | | | | | | | | | |  |
|  | LB | *E. coli* | 0.99 | 0.99 | 75 | 0.0001 | 0.05 | 0.83 |  |  |
|  |  | *C. freundii* | 1.09 | 1.05 | 70 | 0.025 | 2.43 | 0.12 |  |  |
|  | BHI | *E. coli* | 0.91 | 0.88 | 29 | 0.006 | 4.69 | *0.039* |  |  |
|  |  | *C. freundii* | 1.11 | 1.02 | 29 | 0.042 | 41.32 | *< 0.0001* |  |  |
| Lag Time | | | | | | | | | |  |
|  | LB | *E. coli* | 63.87 | 50.41 | 75 | 2485 | 23.97 | *< 0.0001* |  |  |
|  |  | *C. freundii* | 79.91 | 61.40 | 70 | 4414 | 21.50 | *< 0.0001* |  |  |
|  | BHI | *E. coli* | 104.66 | 103.30 | 29 | 9.63 | 0.02 | 0.88 |  |  |
|  |  | *C. freundii* | 152.86 | 132.35 | 29 | 2163 | 8.14 | *0.008* |  |  |
| Stationary Phase Density | | | | | | | | | |  |
|  | LB | *E. coli* | 0.90 | 0.99 | 47 | 0.0779 | 46.91 | *< 0.0001* |  |  |
|  |  | *C. freundii* | 1.05 | 1.05 | 47 | 0.0002 | 0.49 | 0.49 |  |  |
|  | BHI | *E. coli* | 1.21 | 1.118 | 29 | 0.0045 | 12.93 | *0.0012* |  |  |
|  |  | *C. freundii* | 1.17 | 1.21 | 29 | 0.0088 | 120.3 | *< 0.0001* |  |  |
| pH of Spent Media | | | | | | | | | |  |
|  | LB | *E. coli* | 8.91 | 8.92 | 26 | 4.8×10^-5^ | 0.09 | 0.77 |  |  |
|  |  | *C. freundii* | 8.77 | 8.91 | 26 | 0.06 | 39.3 | *< 0.0001* |  |  |
|  | BHI | *E. coli* | 8.11 | 8.40 | 26 | 0.24 | 9.7 | *0.004* |  |  |
|  |  | *C. freundii* | 8.33 | 8.51 | 26 | 0.09 | 1.8 | 0.19 |  |  |

The F and p-values are based on planned comparisons in a single factor ANOVA comparing the ancestor to the evolved populations, which were assayed as whole, polymorphic populations.

1 Degrees of freedom

2 Sums of Squares

3 F statistics

4 p-value, numbers in italics indicate statistical significance
