# Supplementary material for: Mutations in Global Regulators Lead to Metabolic Selection during Adaptation to Complex Environments
Source: PLoS Genet. 2014 Dec 11;10(12):e1004872. doi: 10.1371/journal.pgen.1004872 (PMC4263409; doi:10.1371/journal.pgen.1004872)
Supplement: S3 Table — Estimated media composition based on information provided in BD Diagnostics in Difco & BBL Manual – of Microbiological Culture Media and in BD Bionutrients Technical Manual – Advance Bioprocessing. The composition of individual media components (e.g. tryptone) were listed for the individual components of the media (e.g. tryptone) as % of the dry weight (e.g. total glutamic acid in tryptone is 15%). To estimate the final concentration of the different amino acids in the media, we calculated the content in each component (e.g. tryptone) and accounted for how much of this component was used in the media. The final % for the individual ingredients were summed up over all the components of the media. Note: BHI estimates to not contain “brain heart infusion from solids”. (DOCX) [file pgen.1004872.s012.docx]

| Table S3: Estimated composition of LB Miller and BBL BHI | | |
| --- | --- | --- |
|  | Media (estimated % of dry weight of 1 liter of final media) | |
| Ingredient | LB | BHI |
| Total Nitrogen | 160.25 | 412.87 |
| Total Amino Nitrogen | 71 | 73.57 |
| Alanine free | 32 | 18.8 |
| Alanine total | 60 | 182.8 |
| Arginine free | 29 | 61.75 |
| Arginine total | 63 | 126.15 |
| Asparagine Free | 11 | 3.25 |
| Aspartic acid free | 12 | 3.25 |
| Aspartic acid total | 78.5 | 98.15 |
| Cysteine free | 4 | 4.35 |
| Glutamic acid free | 47 | 7.1 |
| Glutamic acid total | 198 | 163.15 |
| Glutamine free | 2 | 1.45 |
| Glycine free | 7 | 11.45 |
| Glycine total | 32 | 339 |
| Histidine free | 7 | 5.55 |
| Histidine total | 25.5 | 19.3 |
| Isoleucine free | 22 | 10.85 |
| Isoleucine total | 70 | 35.8 |
| Leucine free | 63 | 22.65 |
| Leucine total | 95.5 | 69.2 |
| Lysine free | 64.5 | 42.2 |
| Lysine total | 85 | 68.25 |
| Methionine free | 13 | 6.15 |
| Methionine total | 25 | 15.8 |
| Phenylalanine free | 40 | 24.25 |
| Phenylalanine total | 65 | 51.6 |
| Proline free | 6 | 3.25 |
| Proline total | 76 | 193.45 |
| Serine free | 13.5 | 5.3 |
| Serine total | 30 | 35.1 |
| Threonine free | 12.5 | 3.25 |
| Threonine total | 26 | 19.65 |
| Tryptophan free | 10.5 | 1.8 |
| Tyrosine free | 9 | 10.25 |
| Tyrosine total | 19 | 12.3 |
| Valine free | 28 | 8.55 |
| Valine total | 76.5 | 50.15 |

Media composition based on information provided in BD Diagnostics in Difco & BBL Manual – of Microbiological Culture Media and in BD Bionutrients^TM^  Technical Manual – Advance Bioprocessing. The composition of individual media components (e.g. tryptone) were listed for the individual components of the media (e.g. tryptone) as % of the dry weight (e.g. total glutamic acid in tryptone is 15%). To estimate the final concentration of the different amino acids in % dry weight in one liter of the premixed media, we calculated the content in each component (e.g. tryptone) and accounted for how much of this component was used in the media. The final % for the individual ingredients were summed up over all the components of the media. Note: BHI estimates to not contain “brain heart infusion from solids”.
