# Supplementary material for: Mutations in Global Regulators Lead to Metabolic Selection during Adaptation to Complex Environments
Source: PLoS Genet. 2014 Dec 11;10(12):e1004872. doi: 10.1371/journal.pgen.1004872 (PMC4263409; doi:10.1371/journal.pgen.1004872)
Supplement: S1 Text — Detailed information on the ancestral genomes, phenotypic assays of evolved populations, mutator genotypes, evolution of arcA in populations of a laboratory adapted strain, carbohydrate analyses, subsystem analyses, proteomic analyses and methods. (PDF) [file pgen.1004872.s013.pdf]

Text S1

Mutations in global regulators lead to metabolic selection during adaptation to complex environments

Gerda Saxer[1*], Michael D. Krepps[2,3], Eric D. Merkley[4], Charles Ansong[4], Brooke L. Deatherage Kaiser[4], Marie-Thérèse Valovska[1], Nikola Ristic[5], Ping T. Yeh[1], Vittal Prakash[1], Owen P. Leiser[6], Luay Nakhleh[5,7], Henry S. Gibbons[2], Helen W. Kreuzer[4], Yousif Shamoo[1,7*]

1 Department of BioSciences MS-140, Rice University, 6100 Main Street, Houston, TX 77005, USA

2 United States Army Edgewood Chemical Biological Center, Aberdeen Proving Ground, MD 21010, USA

3 EXCET, Inc, 8001 Braddock Road. Suite 303, Springfield, VA 22151, USA

4 Pacific Northwest National Laboratory, P.O. Box 999, Richland, WA 99352, USA

5 Department of Computer Science, MS-132, Rice University, 6100 Main Street, Houston, TX 77005, USA

6 Center for Microbial Genetics and Genomics, P.O. Box 4073, Northern Arizona University, Flagstaff, AZ 86011, USA

7 Department of Ecology and Evolutionary Biology, Rice University, 6100 Main Street, Houston, TX 77005, USA

Corresponding authors:
gsaxer@rice.edu, shamoo@rice.edu

Ancestral Genomes*: E. coli* RU1 and *C. freundii* RU2

The closely related *E. coli* and *C. freundii* are gamma-proteobacteria and are grouped into the Enterobacteriaceae (Figure S1). Both are facultative anaerobic, Gram-negative commensal bacteria that can be isolated from the human gut flora and are opportunistic pathogens. *C. freundii* is often acquired as nosocomial infections, while *E. coli* infections are typically food-borne. The sequenced genome of *E. coli* RU1 was assembled into 106 contigs at a size of 4.7 MB, with 4565 annotated genes and 63 RNAs. Of the annotated genes, a total of 989 were hypothetical and 2756 (160 of which were hypothetical) were grouped into 578 subsystems (Figure S6, 8a). The genome of *C. freundii* RU2 was assembled into 47 contigs. It was considerably larger than the *E. coli* genome with a size of 5.25 MB, 5068 annotated genes and 78 RNAs. A total of 1047 genes were annotated as hypothetical, and 2950 genes (of which 149 genes were hypothetical) were grouped in 575 subsystems (Figure S6, 8a). Among all the subsystem categories, *E. coli* had significantly more genes than *C. freundii* (relative to the total number of genes annotated) in Phages, Prophages, Transposable Elements and Plasmids (Fisher's exact test: $p = 0.015$), Nucleoside and Nucleotides ($p = 0.048$), Nitrogen Metabolism ($p = 0.044$) and Metabolism of Aromatic Compounds ($p = 0.0042$), while *C. freundii* had significantly more genes that fell in to the subsystems categories of Iron Acquisition and Metabolism ($p<0.0001$) and Motility and Chemotaxis ($p = 0.0013$). Only the last two comparisons remained significant after sequential Bonferroni correction [88].

BBL BHI contains more carbohydrates and amino acids

We used two different premixed media that differed in the amino acid and carbohydrate composition and content: LB Miller (10g tryptone, 5g yeast extract, 10 g

NaCl per liter of water) and BBL BHI (8g brain heart infusion from solids, 5g peptic digest of animal tissue, 16g pancreatic digest of casein, 3g dextrose, 5g NaCl, 2.5g Na2PO4 [89]). To estimate the final concentrations of different amino acids and carbon sources in the media, we used the information published in the BD Bioscience Technical Manual [90] for the individual ingredients and calculated the final composition (in percent dry weight per liter premixed media). Unfortunately, no quantitative composition analysis was available for one of the ingredients of BHI, the brain heart infusion from solids, because this ingredient is not subject to lot quality controls (BD personal communication). Therefore, any estimates for BHI should be considered as minimal estimates. This is especially true for peptides and amino acids, which are likely high in brain heart infusion from solids. Table S3 outlines the estimated final compositions of the two media. The amounts of amino acids, especially arginine, aspartic acid, glycine and proline are considerably higher in BHI than in LB. The addition of dextrose to BHI significantly increased the carbohydrate content in BHI compared to LB.

Phenotypic assays reveal adaptation to the selective environments and underlying genetic variation present in the populations

Both ancestral strains were able to grow well in the selective media, reaching stationary phase densities within 8 hours in LB and about 12 hours in BHI (after 100-fold dilution of stationary phase culture into fresh media). The lag times of the ancestral strains were almost twice as long in BHI than in LB, with very similar growth rates. Stationary phase densities were higher in BHI than in LB (Table S1), consistent with higher nutrient concentrations in BHI.

We hypothesized that adaptation in a relatively flat landscape would progress slowly with small incremental fitness gains. We measured lag time, growth rate and

stationary phase density of the ancestral and evolved populations to assess how the populations adapted to the selective environments. Over the course of the selection experiment, lag time decreased significantly in all LB-evolved populations, but only for *C. freundii* populations evolved in BHI, while maximum growth rate decreased significantly in BHI and showed no changes in LB for both species (Planned comparison between ancestor and evolved populations: Table S1). A comparison between the two species and media showed that growth rate and lag time evolved differently in *E. coli* and *C. freundii* in LB and BHI, as indicated by a full factor ANOVA with Species and selective Media as factors (lag time: Species: $F_{1,206} = 52.4$; Media: $F_{1,206} = 508$, Media×Species: $F_{1,206} = 11.05$, all $p < 0.0001$; growth rate: Species: $F_{1,206} = 120$; Media: $F_{1,206} = 57$; Species×Media: $F_{1,206} = 23.8$, all $p < 0.0001$). The differential adaptation to the two selective media is likely due to the media composition and different dilution rates experienced by the LB- and the BHI-evolved populations. The populations adapted to BHI-evolved for 760 generations and experienced higher daily dilutions (Figure 1), which allowed different genotypes in the population to be in direct competition for longer, resulting in stronger selection for faster growth. It is possible that extending the selection of the LB-evolved populations by another 500 generations would lead to changes in growth rates.

Based on the increased content of carbohydrates and amino acids in BHI, we hypothesized that the populations evolved in BHI should reach higher stationary phase densities than populations evolved in LB. Over the course of the experiment, average density (measured as average OD_{600} between 16 and 24 hours after inoculation) of *E. coli* increased significantly in LB and BHI compared to the ancestor, while average density of *C. freundii* increased significantly only in BHI (LB: $F_{1,47} = 0.48$, $p = 0.48$; BHI: $F_{1,29} = 120.3$, $p < 0.0001$). Overall, average densities were significantly higher in the BHI-evolved populations compared to the LB-evolved populations (*C. freundii*: Media $F_{1,79} =$

810.7, $p < 0.0001$, Block $F_{3,79} = 2.15$, $p = 0.1$; *E. coli*: Media $F_{1,79} = 300$, $p < 0.0001$, Block $F_{3,79} = 4.4$, $p = 0.006$), supporting our hypothesis that the increased nutrient content in BHI would result in higher yield and larger populations at stationary phase.

The *E. coli* populations consistently lost motility, while the *C. freundii* populations retained it. Bacteria that have been cultured in the laboratory are often non-motile [91-93]. We isolated eight randomly chosen single colonies from each evolved population and tested their motility. While both ancestral strains are motile, evolved *E. coli* populations all contained single clones that lost the ancestral swarming ability. While we observed significant differences in loss of motility among the populations ($p < 0.0001$, mixed binary model), loss of motility was consistent across environments in the *E. coli* evolved populations. With the exception of a single clone evolved in LB, all *C. freundii* clones and hence populations retained motility, regardless of the selective environment.

Colony size morphology and size varied considerably in the evolved *E. coli* populations when plated on tetrazolium arabinose plates (Figure 2). The color indicates the cells' ability to utilize arabinose, with white cells being able and dark (red) colonies being unable to use arabinose. To test the genetic variation with and among populations more systematically, we used our test sets of eight single clones from each population and tested them for variations in redox state on methylene blue plates and in exopolysaccharide content on Congo Red plates. While we observed substantial color variation both within and among the BHI-evolved *E. coli* (Figure S2) and all the *C. freundii* populations, we observed a consistent inability of colonies from the LB-evolved *E. coli* populations to grow in the presence of methylene blue. Methylene blue is a known nitric oxide synthetase (NOS) inhibitor [94]. The inability of these colonies to grow could indicate that these colonies are lacking alternative pathways to NOS to deal with oxidative stress. As with methylene blue, we also observed considerable variation within

and among all populations in the content of exopolysaccharides, as indicated by Congo Red staining.

Mutator phenotypes evolved in four *E. coli* populations

Four *E. coli* populations acquired a large number of mutations, suggesting that these populations evolved to become mutators. Indeed, both LB-evolved populations, LB4 and LB11, acquired mutations in the recombination and repair gene *recT* and are the only two populations with mutations in this gene. RecE and RecT work together to promote DNA recombination [95,96]. RecT catalyzes homology-dependent DNA strand exchange and requires ssDNA ends on the linear DNA duplex [96,97]. Population BHI6 had a mutation in the *E. coli* excinuclease ABC subunit A (also known as *uvrC*) and is part of the nucleotide excision repair pathway [98]. BHI10 had a mutation in the DNA polymerase I (*polI*). While all these genes are associated with DNA replication, replication fidelity and repair, it is not certain that these mutations directly contributed to the elevated mutation rates. None of the other populations had mutations in *recT*, *uvrC* or *polI*. We did not observe mutations in the commonly observed *mutL* or *mutS* genes in these four populations. One population, LB2 had a *mutL* mutation, but it did not have as many mutations as the mutator populations.

Mutations in various efflux pumps suggest loss of function and are consistent with the SPANC balance

E. coli and *C. freundii* have multiple copies of the membrane fusion protein of the RND efflux pump, *cmeA*, and of the inner membrane transporter, cmeB. Based on the RAST annotations, our *E. coli* strain has three copies of *cmeA*, and four copies of *cmeB,*

while *C. freundii* only has one copy of *cmeA a*nd five copies of *cmeB*. In the *E. coli* populations, we observed mutations in *cmeA* on contig00003 in four populations (LB5, LB9, LB11 and LB12), all either insertions, deletions or SNPs that resulted in stop codons. A different set of five populations had mutations in *cmeB* on contig00003 (LB1, LB4, LB5, LB7 and LB8), two were substitutions to stop codons, two were insertions and one population had two single nucleotide substitutions. Three BHI-evolved populations acquired mutations in *cmeB:* one population (BHI4) had an insertion and another one (BHI6) had a substitution on contig00044, while one population had an insertion on contig00003. Mutations in *cmeA* and *cmeB* were not as prevalent among the LB-evolved and completely absent among the BHI-evolved *C. freundii* populations. One LB-evolved *C. freundii* population acquired a substitution in *cmeB* on contig00017 (LB25), one had an insertion on contig00001 (LB34) and a third population had an insertion in the RND efflux transporter on contig00005 (LB2). An insertion in *cmeB* on contig00003 evolved in one MA line. The same MA line also acquired an insertion in *cmeA* on contig00027.

Mutations in *arcA* did not evolve in populations of a laboratory strain evolved against the same selection conditions as *E. coli* RU1 and *C. freundii* RU2

We performed our selection experiments with *de novo* isolated strains of *E. coli* and C*. freundii* to be able to use LB and BHI as novel environments. We also evolved twelve replicated populations founded with the two isogenic lab strains *E. coli* B REL606 and *E. coli* B REL607 (six populations each), which only differ in their ability to use arabinose, in LB under the same conditions as the LB-evolved populations. While we did not use whole genome sequencing on these populations, we sequenced the *arcA* locus of thirty single colonies isolated from two evolved populations each (LB13 and LB14). Out of the sixty clones sequenced, not a single one acquired a mutation in *arcA*,

compared to the very abundant *arcA* mutations in the LB-evolved *E. coli* RU1 populations. This difference could be explained by sequence divergence in *arcA* between the two *E. coli* strains as well as the fact that REL606 and REL607 are domesticated laboratory strains. Compared to *E. coli* RU1, *E. coli* B REL606 has three synonymous and one non-synonymous mutations (D11N) in *arcA*. This amino acid change also evolved in our experiment and supports our hypothesis that one single mutation is enough to convey a selective benefit. In addition, this observation further highlights the fact that laboratory adaptation needs to be considered when working with clinical or other *de novo* isolates.

Putrescine secretion and changes in carbohydrates

The increased metabolic rate of the evolved populations resulted in the secretion of putrescine. When we measured putrescine, we also assessed changes in carbohydrates both in the cell extract and in the spent media. Among the carbohydrates tested, only a few changed significantly. In the cell extract, we observed a significant increase of ribose and decreases of mannose and glucose in the evolved populations (Figure S4). In the spent media, only putrescine differed significantly between the ancestor and the evolved populations.

Parallel evolution at the subsystem levels is rare and mainly driven by a few genes

While *arcA* was the only gene that acquired mutations in all evolved LB populations, we also observed several genes with parallel changes among the *C. freundii* populations (Figure 5). The gene encoding the Valine-Glycine Repeat Protein G, *vgrG*, a homolog to the tailspike of bacteriophage T4, acquired mutations in all evolved

C. freundii. Since we used a different filtering method for the *C. freundii* populations than for *E. coli*, it could be possible that these mutations represent systematic errors that did not get discarded during the filtering step. However, we observed a large number of mutations in this gene suggesting that the repeated evolution of mutations in this gene is not due to systematic errors. Adenosylmethionine-8-amino-7-oxononanoate aminotransferase, an enzyme in the biotin to fatty acid biosynthesis pathway acquired mutations in all but one evolved *C. freundii* populations. Unlike mutations in *vgrG*, this mutation could potentially be an error, since the same nucleotide substitution is fixed in all populations it occurs, which is rather unlikely. Nonetheless, one population did not have the same mutation. There are two copies of the same gene annotated in *C. freundii*. The other copy of the gene had a mutation in one LB population.

Parallel evolution among independently evolved populations can also occur along a pathway by affecting different genes of the same pathway in different populations. As such, we would not be able to identify parallel evolution at the level of genes. To test for convergence among independently evolved populations along pathways, we used the three hierarchy levels of the subsystem categories and identified subsystems that were affected repeatedly. At the subsystem level, no clear pattern emerged. Among the *E. coli* populations, no subsystem accumulated mutations in all, or most populations. Leucine biosynthesis acquired mutations in eight LB-evolved lines and no subsystem acquired mutations in eight or more lines among the BHI-evolved populations or the MA lines. The *C. freundii* populations showed more parallel evolution at the subsystem level: All populations acquired mutations in the Type VI secretion system. This is mainly due to mutations in VgrG, the gene that acquired mutations in all evolved *C. freundii* populations. With the exception of one LB-evolved population, all *C. freundii* populations acquire mutations in three different biotin subsystems: Biotin synthesis cluster, biotin biosynthesis and biotin biosynthesis experimental, which was

mostly due to the mutation in the Adenosylmethionine-8-amino-7-oxononanoate aminotransferase assigned to all three subsystems. Lastly, ten populations evolved in LB acquired mutations in the translation termination factors and translation initiation factors. Besides the biotin, protein biosynthesis, and protein secretion systems subcategories already identified above, we also observed mutations in DNA repair in eleven LB-evolved populations, while ten populations had mutations in six other subsystem categories: resistance to antibiotics and toxic compounds; lysine, threonine, methionine and cysteine; glutamine, glutamate, aspartate, asparagine; transcription; regulation and cell signaling; and RNA processing and modification.

At the highest level, the categories, we observed more parallel evolution, but still fewer among the *E. coli* populations than among the *C. freundii* populations (Figure S7). Nine LB-evolved *E. coli* populations acquired mutations in virulence, disease and defense, while seven populations acquired mutations in protein metabolism in LB and BHI. Among the *C. freundii* populations, besides the categories mentioned above, membrane transport, cofactors, vitamins prosthetic group, and protein metabolism, twelve LB-evolved populations also acquired mutations in two more categories: amino acid and derivatives and RNA metabolism. Eleven LB-evolved populations had mutations in carbohydrates; DNA metabolism; regulation and cell signaling, while ten populations had mutations in virulence, disease and defense; and respiration.

To be able to compare changes across species and environments, we identified the number of genes in each subsystem with mutations. We determined how many genes in a subsystem category acquired mutations and calculated a subsystem score for every subsystem, by taking the $\log_2$ ratio as (number of mutations in a subsystem/ genes in subsystem) divided by (number of mutations total / number genes total) and subtracting one. We calculated the subsystem score for each species and environment individually, which allowed us to compare the score among species and environments

(Figure S8). If a subsystem category had more than random mutations, we would get a score larger than zero, indicating that mutations in this system could be adaptive. If a subsystem category had fewer genes with mutations than expected, we would get a score smaller than zero, indicating more constraints on genes in these subsystems. Subsystems that acquired mutations in response to adaptation should have a similar subsystem score either for both species for environments. We see a few surprising spikes in subsystem categories that acquired more mutations than expected such as dormancy and sporulation in BHI-evolved *E. coli* populations or virulence, disease and defense in BHI-evolved *C. freundii* populations. However, with the exception of phages, prophages, transposable elements and plasmids, where we observed fewer mutations in BHI and more than expected mutations in LB in both species, no clear pattern emerges. The lack of a clear pattern is consistent with the observed mutations in the global regulators *arcA* and *rpoS*, which act like a short cut to the accumulation of lots of different mutations necessary to get these global effects. While *rpoS* was grouped in RNA metabolism, *arcA* was not associated with any subsystem category.

Proteomics

Across 39 independent single dimension (1-D) LC-MS analyses of the *E. coli* ancestor sample and the twelve corresponding evolved population samples, we identified 4469 unique peptides. The PCA plot of the peptide abundances across the ancestor and evolved population samples showed a clear separation between the two groups (Figure S9A). This was further supported by a Pearson correlation analysis, which also showed a clear distinction between the ancestor and evolved population samples (Figure S9B). These 4469 peptides corresponded to 488 proteins, with the requirement each protein was identified by at least 2 unique peptides. Quantitative

analysis of the 488 proteins revealed 166 proteins that were significantly different ($p<0.01$; log_2-fold change >± 0.7). Of those 58 proteins were significantly down-regulated in the evolved populations relative to the ancestor samples and 108 proteins were significantly up-regulated in the evolved population samples relative to the ancestor samples (Figure 6, Table S2).

Material and Methods

Phenotypic Assays

Assays were performed either at the population level or the single colony level. We isolated eight randomly chosen single colonies from every population and froze them at -80°C for subsequent use. Cells or populations were grown in their selective media (LB or BHI) and plated on agar plates made with their selective media, unless otherwise stated.

Growth assays and average stationary phase density: To assess fitness changes, we measured growth rates over 24 hours by diluting stationary phase cultures 100-fold into fresh LB media and measuring OD every 5 minutes over 24 hours. Maximum growth rate and lag time was analyzed following Walkiewicz et al. [74]. Average density was assessed as the average OD_{600} over 16-24 hours after inoculation.

Motility Assay: Single colonies were grown to stationary phase in their selective media, diluted 1000-fold and plated on LB + 0.25% DIFCO agar or BHI + 0.25% DIFCO agar depending on their selective environment to assess their motility and swarming ability. The soft agar plates were incubated at room temperature for two days, before we scored the plates and determined whether a colony was swarming or not. Each assay was performed in triplicate. We used a mixed binary model with block and population as mixed factors to test for differences in loss of migration among the populations for each

environment separately and tested for differences between selective environments by analyzing both environments combined. Only data from *E. coli* were analyzed as only one *C. freundii* isolate lost motility.

Variation in colony size morphology: Stationary phase populations were plated at low density on tetrazolium arabinose (TA) plates and incubated for 48 hours at 37°C.

Redox state and exopolysaccharide content: To assess the genetic variation within populations, we plated 8 single colonies from every population on LB or BHI agar plates supplemented with 0.065g/liter methylene blue [99], or with 0.15g/liter Congo Red. Methylene blue changes color in response to redox conditions and pH, while Congo Red stains exopolysaccharides.

Preparation of samples for proteomics analysis

Protein was extracted by re-suspending cell pellets in 50 µL lysis buffer [6 M urea (Sigma U-0631) and 14.3 mM 2-mercaptoethanol (Sigma (M6240) in 100 mM triethylammonium bicarbonate (TEAB), pH 9 (Sigma T7408)]. Samples were then incubated for one hour at 60°C with shaking at 600 rpm on a thermomixer. Insoluble cell material was removed by brief centrifugation. To the supernatant, 400 µL of 100 mM TEAB was added followed by 5 µL of 375 mM iodacetamide (Pierce 90034) in 100 mM TEAB. Samples were incubated for 30 minutes in the dark. Proteins were digested with 2.5 µg of trypsin (Promega V5280) for 14 hours at 37°C with gentle shaking. Solid phase extraction (SPE) was performed with a vacuum manifold using Strata C-18 T columns and following the manufacturer's protocol. Briefly, 1 mL of 100% methanol (Sigma 675415) was added to activate the resin, followed by a conditioning rinse of 1 mL 0.1% trifluoroacetic acid (TFA, Sigma T6399) water, then addition of the samples in 100 mM TEAB. The samples were washed with 0.1% TFA water, and eluted with 80% acetonitrile (Simga 675415) in 0.1% TFA water into clean low protein-binding 1.5 mL microfuge

tubes (Fisher 02-681-320). Samples were dried down to near completeness (~ 5 µL) with an Eppendorf Vacufuge plus. Peptides were resuspended with 0.1% formic acid water (Suprapur EMD 11670) and the concentration was adjusted to 1 mg/mL as measured using the BCA assay (Pierce 23225). The samples were then transferred to high performance liquid chromatography (HPLC) vials with 250 µL inert glass inserts and capped with screw caps (Agilent 5182-0715, 5181-8872, 5182-0723). Samples were stored at -20°C prior to analysis.

Liquid chromatography-mass spectrometry measurements for proteomics

Digested peptide samples were injected onto an Agilent Infinity 100 HPLC system. The column was a 40 cm long x 105 µM inner diameter fused silica capillary packed with 5 µM C18 resin (Phenomenex, Torrance, California). 1 µL aliquots (total mass ~1 µg) were injected and subjected to the following gradient 160-minute gradient: 100% Solvent A for 10 minutes; 0% B to 7.5% B over 1 minute; 7.5% B to 45% B over 110 minutes; 45% B to 95% B over 2 minutes; 95% B for 10 minutes, 95% B to 0% B over 4 minutes, and 100% A for 20 minutes. Solvent A was 5% acetonitrile/0.1% formic acid, and Solvent B was 95% acetonitrile/0.1% formic acid. Blanks consisting of 5 µL injections of isopropyl alcohol were run (with a shorter gradient) between samples to minimize column carry-over.

To minimize statistical impacts to the experiment due to run order or adverse instrumental events, samples were grouped into batches such that experimental factors were balanced across each batch. Each batch of samples was run in a block with randomized run order. Each block was repeated a total of three times with a different random run order each time. To monitor the quality of the chromatographic separation, standards were run before and after each block. The standard was a tryptic digest of

ovalbumin, bovine serum albumin, bovine αS1-casein, and bovine lactalbumin (all from Sigma) at equal mass concentrations.

The HPLC was coupled to a Thermo Scientific LTQ Orbitrap XL mass spectrometer via a custom electropspray emitter consisting of an etched fused silica capillary [100]. The MS was operated in data dependent “high-low” mode with a high-resolution (*R*=30,000) precursor scan collected in the Orbitrap followed by collision-induced dissociation (CID) fragment scans of the top 7 most intense precursors collected in the ion trap. Data dependent acquisition parameters were: CID fragmentation normalized collision energy 35%; monoisotopic precursor selection enabled; dynamic exclusion repeat count 2, repeat duration 30 s, exclusion list size 250, exclusion list duration 180 s.

Proteomics Data Analysis utilizing AMT tag approach

In brief, this approach utilizes tandem mass spectrometry (MS/MS) to generate a reference peptide database (accurate mass and time tag database; AMT tag database) of observed peptides, their associated theoretical masses, and LC elution times (normalized). This database is utilized to assign peptide sequences to ion current (relative abundance) information of peptides measured using high-resolution, high mass measurement accuracy mass spectrometry (LC-MS)[86].

Generated MS/MS spectra were searched using the MSGF algorithm [101] against the *E. coli* RU1 translated genome sequence. Identified peptides of at least six amino acids in length having MS-GF score ≤1E-10, which corresponds to an estimated FDR <1% at the peptide level, were used to generate an AMT tag database. This database comprises the observed peptides, their associated theoretical masses, and normalized LC elution times.

Orbitrap spectra were deisotoped using the software tool Decon2LS [102] after which mass and elution time features were identified and matched with VIPER [103] to peptides stored in the *E. coli* RU1 AMT tag database within mass measurement accuracy and elution time accuracy cut-offs of <2 ppm and <2%, respectively. Measured arbitrary abundance for a particular peptide was determined by integrating the area under each LC–MS peak for the detected feature matching to that peptide. Matched features from each Orbitrap analysis (dataset) were then filtered on a false discovery rate (FDR) of ≤5%; the FDR associated with the AMT tag proteomics approach is calculated using STAC (Statistical Tools for AMT tag confidence), a statistical algorithm for assigning confidence to matched mass and elution time features [104]. Relative peptide abundance measurements in technical replicates were scaled and normalized in DAnTE [87]. Normalized peptide abundance values were then rolled up to proteins using RRollup [87]; a minimum of five peptides was required for the Grubb's test, with a *p*-value cutoff of 0.05. Only peptides unique in identifying a single protein were utilized to estimate protein abundances. Additionally, proteins represented by <2 unique peptides were removed. ANOVA analyses were applied to protein abundance data sets (*p*-value ≤0.01) to identify statistically significant differences in protein expression levels.

GC-MS analysis of putrescine and carbohydrates

Putrescine secretion was measured with changes in carbohydrates. *C*arbohydrate profiles were determined by hydrolyzing cells to liberate monosaccharides, derivatizing the sugars to volatile forms, and analyzing the derivatives by gas chromatograpy/mass spectrometry (GC-MS). We used the alditol acetate method to prepare the carbohydrates for analysis [105]. Sugar monomers (e.g., glucose, mannose, glucosamine, etc.) were liberated from any cellular polymers using 2 N sulfuric acid hydrolysis at 100°C for 3 hours in a nitrogen atmosphere. Following hydrolysis, samples

were neutralized with a liquid-liquid extraction using N,N dioctyl methylamine base in chloroform, and hydrophobic contaminants were removed by solid phase extraction with 1 mL C-18T cartridges (Phenomenex, Torrence, CA). Reduction of aldehydes to their alditol form was performed using sodium borodeuteride, and residual borodeuteride was removed as tetra methyl borate gas by addition of methanol-acetic acid under streaming nitrogen. Derivatization of reduced sugars was performed by addition of acetic anhydride at 100°C for 13-16 hours to form acetate derivatives for GC-MS analysis. Carbohydrate concentrations were calculated based upon a ratio of peak areas between known amounts of external and internal standards, which were analyzed as separate samples in each batch. The amount of each derivatized sugar in a biomass sample was determined by a ratio to the internal standard, adjusting for the relative response determined from the standard mixture and then calculating a mass per sample volume.

References

88. Rice WR (1989) Analyzing Tables of Statistical Tests. Evolution 43: 223-225.

89. Zimbro MJ, Power DA, Miller SM, Wilson GE, Johnson JA (2009) Difco & BBL Manual - Manual of Microbiological Culture Media: BD Diagnostics - Diagnositc Systems, Sparks, MD.

90. Bioscience B (2006) BD Bionutrients ™ Technical Manual - Advanced Bioprocessing. USA. 68 p.

91. Kearns DB, Losick R (2003) Swarming motility in undomesticated Bacillus subtilis. Molecular Microbiology 49: 581-590.

92. Dinsdale EA, Edwards RA, Hall D, Angly F, Breitbart M, et al. (2008) Functional metagenomic profiling of nine biomes. Nature 452: 629-U628.

93. Patrick JE, Kearns DB (2009) Laboratory Strains of Bacillus subtilis Do Not Exhibit Swarming Motility. Journal of Bacteriology 191: 7129-7133.
94. Mayer B, Brunner F, Schmidt K (1993) Inhibition of Nitric-Oxide Synthesis by Methylene-Blue. Biochemical Pharmacology 45: 367-374.
95. Clark AJ, Sharma V, Brenowitz S, Chu CC, Sandler S, et al. (1993) Genetic and Molecular Analyses of the C-Terminal Region of the Rece Gene from the Rac Prophage of Escherichia-Coli K-12 Reveal the Rect Gene. Journal of Bacteriology 175: 7673-7682.
96. Hall SD, Kolodner RD (1994) Homologous Pairing and Strand Exchange Promoted by the Escherichia-Coli Rect Protein. Proceedings of the National Academy of Sciences of the United States of America 91: 3205-3209.
97. Thresher RJ, Makhov AM, Hall SD, Kolodner R, Griffith JD (1995) Electron-Microscopic Visualization of Rect Protein and Its Complexes with DNA. Journal of Molecular Biology 254: 364-371.
98. Kisker C, Kuper J, Van Houten B (2013) Prokaryotic Nucleotide Excision Repair. Cold Spring Harbor Perspectives in Biology 5.
99. Hulanicki A, Głab S (1978) Redox indicators. Characteristics and applications. Pure and Applied Chemistry 50: 463-498.
100. Kelly RT, Page JS, Luo Q, Moore RJ, Orton DJ, et al. (2006) Chemically etched open tubular and monolithic emitters for nanoelectrospray ionization mass spectrometry. Anal Chem 78: 7796-7801.
101. Kim S, Gupta N, Pevzner PA (2008) Spectral probabilities and generating functions of tandem mass spectra: a strike against decoy databases. J Proteome Res 7: 3354-3363.
102. Jaitly N, Mayampurath A, Littlefield K, Adkins JN, Anderson GA, et al. (2009) Decon2LS: An open-source software package for automated processing and

visualization of high resolution mass spectrometry data. BMC Bioinformatics 10: 87.

103. Monroe ME, Tolic N, Jaitly N, Shaw JL, Adkins JN, et al. (2007) VIPER: an advanced software package to support high-throughput LC-MS peptide identification. Bioinformatics 23: 2021-2023.

104. Stanley JR, Adkins JN, Slysz GW, Monroe ME, Purvine SO, et al. (2011) A statistical method for assessing peptide identification confidence in accurate mass and time tag proteomics. Anal Chem 83: 6135-6140.

105. Fox A, Morgan SL, Gilbart J (1989) Preparation of alditol acetates and their analysis by gas chromatrography (GC) and mass spectrometry (MS). In: Biermann CJ, McGinnis GD, editors. Analysis of Carbohydrates by GLC and MS. Boca Raton, FL: CRC Press. pp. 87-117.
